# Oscillatory-Quality of sleep spindles links brain state with sleep regulation and function

**DOI:** 10.1126/sciadv.adn6247

**Published:** 2024-09-06

**Authors:** Cristina Blanco-Duque, Suraya A. Bond, Lukas B. Krone, Jean-Phillipe Dufour, Edward C. P. Gillen, Ross J. Purple, Martin C. Kahn, David M. Bannerman, Edward O. Mann, Peter Achermann, Eckehard Olbrich, Vladyslav V. Vyazovskiy

**Affiliations:** ^1^ Department of Physiology, Anatomy and Genetics, University of Oxford, Sherrington Building, Sherrington Rd, Oxford OX1 3PT, UK.; ^2^ Department of Brain and Cognitive Sciences, Massachusetts Institute of Technology, 43 Vassar St, Cambridge, MA 02139, USA.; ^3^ UK Dementia Research Institute at UCL, University College London, WC1E 6BT London, UK.; ^4^ University Hospital of Psychiatry and Psychotherapy, University of Bern, Bolligenstrasse 111, 3000 Bern 60, Switzerland.; ^5^ Astrophysics Group, Cavendish Laboratory, J.J. Thomson Avenue, Cambridge CB30HE, UK.; ^6^ Astronomy Unit, Queen Mary University of London, Mile End Road, London E14NS, UK.; ^7^ School of Physiology Pharmacology and Neuroscience, University of Bristol, Bristol BS8 1TD, UK.; ^8^ Department of Experimental Psychology, University of Oxford, Oxford OX2 6GG, UK.; ^9^ Institute of Pharmacology and Toxicology, University of Zurich, Winterthurerstrasse 190, Zurich CH-8057, Switzerland.; ^10^ Max Planck Institute for Mathematics in the Sciences, Inselstraße 22, 04103 Leipzig, Germany.; ^11^ Sleep and Circadian Neuroscience Institute, University of Oxford, Sherrington Rd, Oxford OX1 3QU, UK.; ^12^ The Kavli Institute for Nanoscience Discovery, University of Oxford, Sherrington Rd, Oxford OX1 3QU, UK.

## Abstract

Here, we characterized the dynamics of sleep spindles, focusing on their damping, which we estimated using a metric called oscillatory-Quality (o-Quality), derived by fitting an autoregressive model to electrophysiological signals, recorded from the cortex in mice. The o-Quality of sleep spindles correlates weakly with their amplitude, shows marked laminar differences and regional topography across cortical regions, reflects the level of synchrony within and between cortical networks, is strongly modulated by sleep-wake history, reflects the degree of sensory disconnection, and correlates with the strength of coupling between spindles and slow waves. As most spindle events are highly localized and not detectable with conventional low-density recording approaches, o-Quality thus emerges as a valuable metric that allows us to infer the spread and dynamics of spindle activity across the brain and directly links their spatiotemporal dynamics with local and global regulation of brain states, sleep regulation, and function.

## Teaser

Oscillatory strength of sleep spindles is a key variable reflecting the network dynamics and functional role of spindles.

## INTRODUCTION

Brain networks have an intrinsic capacity to generate and sustain a wide range of neural oscillations, which are thought to be a fundamental basis for cognition and behavior. Among these rhythms are sleep spindles, which are traditionally defined as bursts of oscillatory brain activity with frequencies of ~10 to 15 Hz and durations of 0.5 to 3 s, observed during non–rapid eye movement (NREM) sleep (*1*). These oscillations arise in the reticular nucleus of the thalamus (*2*, *3*) and express either locally or across widespread thalamo-cortical networks, exhibiting notable variability in their frequency, shape, and topography (*4*–*8*). An increasing body of research using multi-site recordings of brain activity alongside time-frequency analyses has focused on examining spindle variability in terms of density, amplitude, and frequency across the brain, aiming to understand their network dynamics and functional role (*8*–*10*). Furthermore, studies indicate a key role of spindles in brain-wide dynamics during sleep, with emerging evidence suggesting that they support offline information processing (*11*–*15*) or protect sleep from sensory disruption (*16*–*21*). However, contradictory findings have arisen, challenging these conclusions (*22*).

In addition to density, amplitude, frequency, and topography, there exist other fundamental characteristics of brain oscillations, such as their damping, that have not been thoroughly investigated but, we argue, are posited to enhance our understanding of the origin and function of sleep spindles. Damping is a metric frequently used in physics and engineering that measures the decay in the amplitude of an oscillation over time (*23*), and therefore reflects levels of oscillatory strength and stability (*24*). Damping has recently proven to be a useful metric for the detection of oscillatory brain activity, like sleep spindles or alpha bursts, which are believed to occur in the form of discrete events (*25*). However, the potential relevance of the oscillatory strength of brain activity for defining network dynamics or functional role has not been investigated.

Here, we aimed to characterize the variability of spindles in terms of oscillatory strength and investigate the physiological and functional meaning of this variability. To this end, we developed a method to quantitatively measure the strength of spindle oscillations, which is based on a time-frequency analysis, utilizing autoregressive (AR) modeling of short segments of electroencephalogram (EEG) signals introduced earlier by Olbrich and Achermann (*25*) for human EEG. Here we extended this method not only to allow the detection of spindles but also to characterize properties of spindle events based on their damping. As this metric allows us to quantitatively describe the variability of spindles in terms of their oscillatory strength, we call it the oscillatory-Quality (o-Quality, oQ). Our approach does not require band-pass filtering, i.e., the result does not depend on whether the spindle frequency is at the center or close to the borders (cutoff frequencies) of the specified pass band of the filter, and uses damping—a less noisy readout of oscillatory strength of the signal than its amplitude.

Next, we characterize the variability of spindles in terms of oscillatory strength across the mouse brain under different experimental conditions. Specifically, we combine multi-site recordings of cortical LFPs and neuronal activity and estimate the o-Quality of spindles recorded across cortical regions and layers, during spontaneous sleep and after sleep deprivation (SD), as well as in wild-type (WT) and transgenic mice lacking the GluA1 subunit of the α-amino-3-hydroxy-5-methyl-4-isoxazolepropionic acid (AMPA) receptor, which were previously found to present deficits in EEG spindles (*26*). Last, we apply our technique to advance our understanding of the neurophysiological and functional role of sleep spindles. Specifically, we investigate whether the o-Quality of spindles is related to sensory responsiveness to auditory stimulation during sleep.

Invariably, we find that it is not merely the all-or-none incidence of spindles that matters, but their o-Quality emerges as a key variable reflecting the network dynamics and functional role of spindles. Specifically, we show here that the o-Quality of spindles reflects their network synchrony, with low–o-Quality spindles showing high incidence and locality, and high–o-Quality spindles showing low incidence but extending across widespread cortical areas. We also show that GluA1-mediated neurotransmission is essential for the large-scale network synchronization of spindles. Last, our results support previous findings suggesting that spindles protect sleep from sensory disruption and further demonstrate that the o-Quality and network synchrony of spindles supports this functional role.

## RESULTS

### Spindles show substantial variability in their oscillatory strength

**Fig. 1.**
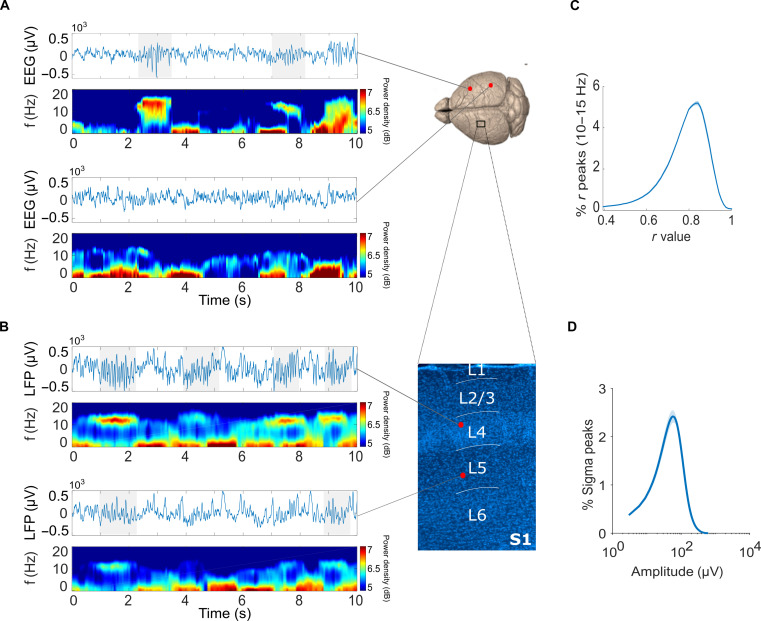
Spindles show a substantial variability in their oscillatory strength. (**A** and **B**) Ten-second signal segments and respective spectrograms for corresponding signals recorded simultaneously from the frontal EEG [(A), top] and occipital EEG [(A), bottom] electrodes, or LFP recorded from layer 4 of S1 [(B), top] and layer 5 of S1 [(B), bottom]. Spectrograms are color-coded on a logarithmic scale. (**C**) Distribution of the maximum *r* value across poles with frequencies (*𝑓_𝑘_*) between 10 and 15 Hz for an LFP signal recorded from layer 4 in S1. (**D**) Peak sigma (10 to 15 Hz) amplitude distribution for the same LFP signal used in (C) (layer 4 in S1). In (C) and (D), line = mean across seven mice. Shaded area = SEM. EEG, electroencephalogram; LFP, local field potential; S1, primary sensory cortex; SEM, standard error of the mean.

We performed continuous electrophysiological recordings of the EEG from frontal, parietal, and occipital regions, combined with multichannel local field potentials (LFPs) from the primary somatosensory (S1, *n* = 21 mice) or primary motor (M1, *n* = 7 mice) cortices (fig. S1, A to F), in undisturbed freely moving mice, entrained to a 12-hour light/12-hour dark cycle. As expected, all mice slept predominantly during the light phase, of which they spent 84.3 ± 1.28% of time in NREM sleep (fig. S1B). Visual inspection of the signals confirmed the occurrence of bursts of oscillatory activity at the spindle frequency (10 to 15 Hz) in both the EEG and the LFP signals, which showed marked variability in their occurrence and characteristics across time, cortical layers, and cortical areas (Fig. 1, A and B). For example, within a specific location (i.e., a specific recording channel), some events were clearly distinct from background activity, while other events were barely discernible. Furthermore, some putative spindle events were prominent across widespread cortical areas (i.e., several LFP channels or different brain regions), while others were readily observed only in one or two channels.

This variability in spindle-like activity is well known to researchers and was also confirmed in our dataset (Fig. 1, C and D). For example, using AR modeling and plotting the inverse damping distribution *r* of oscillators (larger *r* values correspond to lower damping) with frequencies between 10 and 15 Hz during NREM sleep in LFP signals recorded from S1 revealed that oscillatory activity between 10 and 15 Hz shows a continuous variation in its damping (Fig. 1C). This was consistent with the observation of a continuous distribution of LFP amplitudes after band-pass filtering of LFP signals from NREM sleep between ~10 and 16 Hz (Fig. 1D)—a procedure widely used in the literature to detect spindle events (*27*).

The key premise for this study was the notion that focusing merely on quantitative measurements of spindle activity, such as their incidence, does not consider the strength of individual spindle events (i.e., how “strong” the oscillatory activity in the spindle frequency range is during a specific spindle event). This is not merely a methodological issue that can be satisfactorily addressed with the advent of more sophisticated approaches for threshold optimization. Instead, it highlights the likely possibility that the variability in spindle characteristics has an important meaning beyond what the scrutiny of arbitrarily defined events can provide. We propose that the variability of spindle activity in terms of oscillatory strength represents a fundamentally important dimension that can help to clarify the underlying neurophysiological mechanisms and function of spindles.

### The o-Quality is a quantitative metric of spindle activity strength

**Fig. 2.**
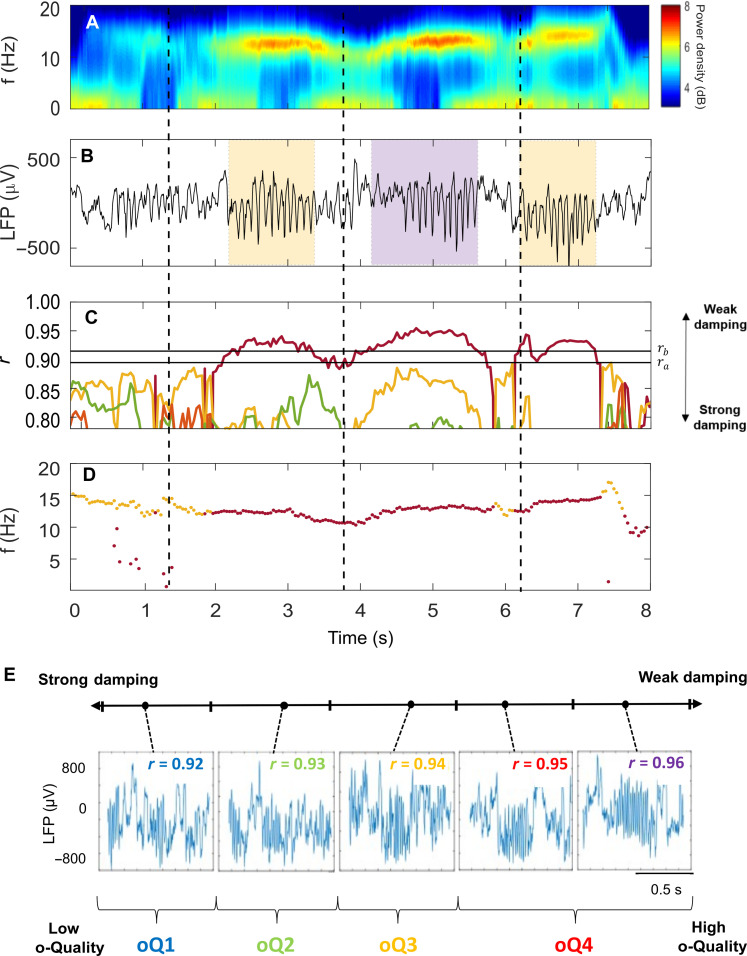
The o-Quality: a quantitative metric of spindle activity strength. (**A**) Spectrogram of an 8-s segment of LFP recording from S1 in a representative mouse. Spectra are color-coded on a logarithmic scale. (**B**) Eight-second segment of LFP data during NREM sleep showing a sequence of detected spindle events, highlighted by shaded colored boxes. The yellow boxes indicate spindle events whose max 𝑟 values reached 0.94, while the purple box indicates a spindle event whose max 𝑟 value reached 0.95. (**C**) Absolute 𝑟 values for the four poles estimated by the AR(8) model. Each pole is represented with a different color. The black horizontal lines represent the upper threshold used for detection of oscillatory events (i.e., *𝑟_𝑏_* = 0.92) and the lower threshold (*𝑟_𝑎_* = 0.90) used to merge or separate consecutive oscillatory events. (**D**) Frequencies *𝑓_𝑘_* of the poles with lowest damping. (**E**) Examples of spindle events with different levels of damping (i.e., different maximum *r* values). The maximum 𝑟 value for each detected spindle was used to group spindles into four o-Quality groups (oQ1 to oQ4) such that strong-to-weak damping corresponds to low to high o-Quality. LFP, local field potential; S1, primary sensory cortex; AR, autoregressive.

At the core of our approach is an algorithm that detects oscillatory events in brain signals based on their damping (*25*), a measurement used to parameterize oscillatory strength (*24*). We applied this model to (i) detect spindles on mouse EEG and LFP signals and (ii) to characterize spindles based on varying levels of damping. The algorithm consists of fitting an AR model of order *p* = 8 to 1-s segments of LFP and EEG signals, shifted by one sampling interval throughout the data, which results in a model with a maximum of *p*/2 oscillators with damping and frequency varying in time (Fig. 2, A to D). The coefficients of the AR(8) model are used to calculate an *r_k_* coefficient (with *k* indicating the corresponding modeled oscillator), whose negative logarithm is proportional to the damping constant; therefore, *r_k_* = 1 means no damping and *r_k_* = 0 means maximum damping (see Materials and Methods). When the signal is dominated by rhythmic activity like a spindle event (Fig. 2, A and B), this activity is reflected by a decrease in damping and hence an increase in *r_k_* (Fig. 2C), in an oscillator with the corresponding frequency *f_k_* (Fig. 2D). Events are detected when the 𝑟_𝑘_ of an oscillator with frequency *f_k_* exceeds a predefined detection threshold (*r_b_*),^46,50^ and detections are tagged with their respective maximum *r_k_* value and the *f_k_* where 𝑟 is maximum (Fig. 2E). Most events detected with this approach during NREM sleep were clustered in the traditionally accepted spindle frequency range in rodents (10 to 15 Hz) and in the delta range (fig. S2A). For subsequent analyses, we selected events with tagged *f_k_* between 10 and 15 Hz.

In engineering and physics, the level of damping in oscillatory systems (*23*, *24*) is parameterized in terms of a quality factor (*23*). In analogy to this metric, we defined an index to parameterize the damping level (i.e., oscillatory strength) in brain oscillations, which we refer to as o-Quality (oQ). Specifically, we used the maximum 𝑟 value detected for each event to group spindles into four o-Quality groups (oQ1 to oQ4) such that strong-to-weak damping corresponds to low to high o-Quality. These groups were set such that spindles with a maximum 𝑟 value between 0.92 ≤ *r* < 0.93, 0.93 ≤ *r* < 0.94, 0.94 ≤ *r* < 0.95, and 0.95 ≤ *r* would be classified as oQ1, oQ2, oQ3, and oQ4, respectively (Fig. 2E). Notably, apart from taking into consideration the variability of spindles in their oscillatory strength, this approach does not assume any specific oscillatory waveform and does not require signal filtering in any specific frequency band. This circumvents the potential signal distortion that band-pass filters may generate (*28*) and makes this approach suitable for spindle analysis in other animal species and humans with different conditions that may add variability to spindle features.

We observed that the o-Quality of sleep spindles showed a strong positive relationship with their duration (*F*_3,18_ = 674.7, *P* < 0.0001) and frequency (*F*_3,18_ = 21.34, *P* < 0.001) (fig. S2). Spindles with low o-Quality, however, occurred at a significantly higher rate (*F*_1.3,7.5_ = 137.9 GG, *P* < 0.0001) than high–o-Quality spindles (fig. S2). We further observed that, on average, spindles with high o-Quality showed a higher power in the spindle frequency range than low–o-Quality spindles (fig. S2F), possibly reflecting a generally higher amplitude of high–o-Quality events. As signal amplitude is a popular metric traditionally used for detecting individual spindle events, we next systematically investigated the relationship between spindle o-Quality and their corresponding amplitude, measured as the maximum value of the Hilbert transform of the band-pass–filtered signal between 10 and 15 Hz during individual spindle events (figs. S2C and S3). Plotting *r* values against corresponding spindle amplitudes revealed, however, only a weak positive relationship (figs. S2C and S3), with <10% of the variance explained. This suggests that damping of spindle oscillations does not merely mirror signal amplitude, which varies greatly across spindle events irrespective of their oscillatory strength. To further explore the association between spindle o-Quality and spindle amplitude, we subdivided spindle events detected with the two approaches—(i) the AR model as described above and (ii) a traditional amplitude-based algorithm (see Materials and Methods)—into four amplitude categories, namely, Amp1 to Amp4 (where Amp1 = lowest amplitude and Amp4 = highest amplitude), and evaluated their relationship with damping for each individual animal (fig. S3, A to D). This analysis showed that, on average, most spindle events clustered within a very narrow range of o-Quality values, regardless of their amplitude, and, regardless of the approach for spindle detection, the amplitude of spindles was only weakly related with spindle o-Quality (the AR model: *F*_3,18_ = 31.7, *P* < 0.01; the amplitude-based approach: *F*_3,18_ = 23.05, *P* < 0.01). This suggests that o-Quality of sleep spindles represents a metric that does not merely reflect spindle amplitude, used traditionally for spindle detection, and therefore may offer a fresh and unique perspective on addressing the dynamics and function of sleep spindles.

### The spatial dynamics of sleep spindles are reflected in their o-Quality

**Fig. 3.**
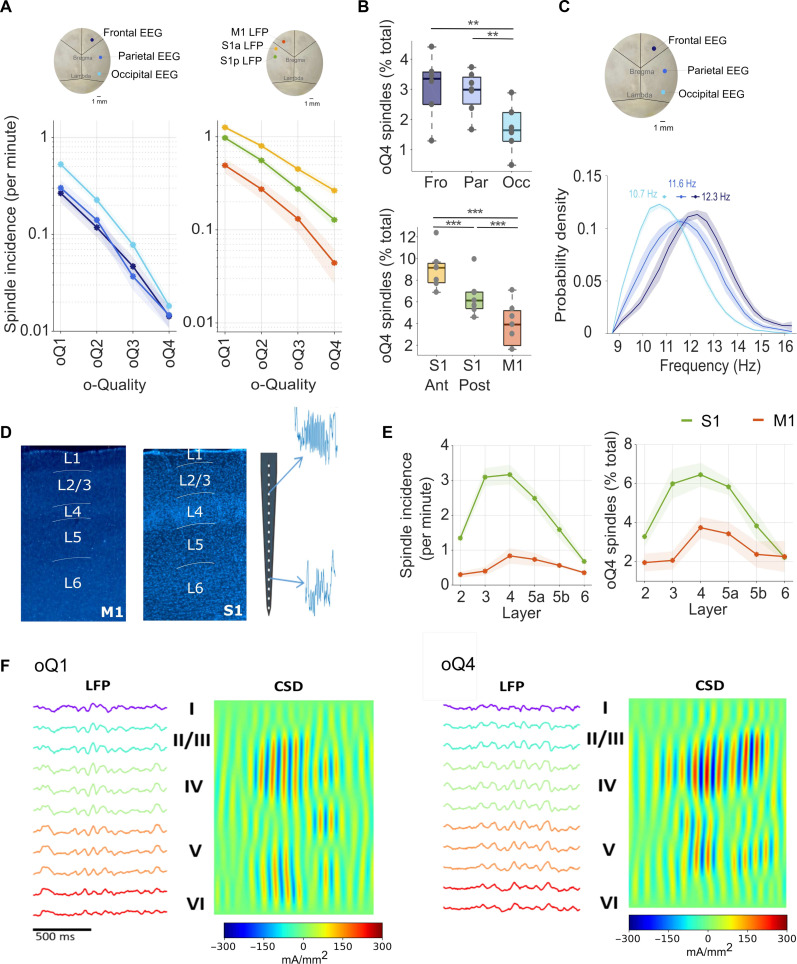
The o-Quality reflects spatial dynamics of sleep spindles. (**A**) Incidence (per minute of NREM sleep) of spindles detected in EEG (frontal, parietal, and occipital) and LFP (anterior S1, posterior S1, and M1) derivations as a function of spindle o-Quality. Dots = mean across mice; shaded areas = SEM. (**B**) Number of high–o-Quality (oQ4) spindles as a percent of total spindles detected in EEG (frontal, parietal, and occipital) and LFP (anterior S1, posterior S1, and M1) derivations. For boxplots: black lines = mean across mice, boxes = SEM, whiskers = 95% confidence intervals, and dots = individual values for each mouse. **P < 0.01, ***P < 0.001. (**C**) Frequency (in hertz) distribution for spindles detected in different EEG derivations (frontal, parietal, and occipital). Lines = mean across mice; shaded areas = SEM. (**D**) Histological verification of probe location across cortical layers in M1 and S1. Illustrations showing examples of spindle events detected in cortical layers 4 and 6 of S1 (right). (**E**) Mean spindle incidence per minute (left) and percentage of detected high–o-Quality (oQ4) spindles (percentage of total number of spindles; right) across different cortical layers of S1 and M1 cortices. (**F**) Example o-Quality 1 and o-Quality 4 spindles with LFP and corresponding current source density (CSD; red, current source; blue, current sink) signal of primary somatosensory cortex. Layer centroids are marked by roman numerals. EEG, electroencephalogram; LFP, local field potential; S1, primary sensory cortex; M1, primary motor cortex; SEM, standard error of the mean.

First, we posited that if the variability across spindle events in terms of o-Quality is biologically meaningful, it should be related to their spatiotemporal dynamics. As previous studies indicate that incidence and frequency of sleep spindles varies as a function of brain region and cortical area, we hypothesized that the o-Quality of spindles will also show topographical gradients. Consistent with this prediction, we found that the incidence of EEG spindles with different o-Quality (derivation × o-Quality interaction: *F*_6,54_ = 4.96, *P <* 0.01) (Fig. 3A, left), as well as the proportion of high–o-Quality events detected on EEG signals (*F*_2,20_ = 6.45, *P* < 0.01) (Fig. 3B, top), varied between cortical regions. Generally, across the frontal, parietal, and occipital cortices, EEG spindles with a higher o-Quality index were more prominent in more anterior cortical areas (Fig. 3B, top).

Consistent with the finding of a positive but weak relationship between intra-spindle frequency and o-Quality (fig. S2D), we observed that the predominant frequency of EEG spindle events varied among cortical areas, with slowest spindles occurring in the occipital cortex (*F*_2,20_ = 16.38, *P* < 0.001) (Fig. 3C). These results are in line with previous mouse EEG studies (*29*). Conversely, human studies have reported that spindles show an anteroposterior increase in their frequency (*5*, *8*, *30*).

Likewise, the distribution of LFP spindles as a function of their o-Quality (oQ1 to oQ4) varied between cortical regions (derivation × o-Quality interaction *F*_2.56,54_ = 19.81 GG, *P* < 0.01) (Fig. 3A, right). Specifically, we found that spindles recorded with LFP electrodes from two locations within S1 were of higher o-Quality in more anterior locations and overall showed higher o-Quality than spindles recorded with LFP electrodes from M1 (*F*_2,20_ = 13.38, *P* < 0.001) (Fig. 3A, right, and Fig. 3B, bottom). These results suggest that the oscillatory strength of spindles is not homogeneous across the cortex but shows distinct topographic gradients, consistent with established morphological and functional differences between cortical areas.

In contrast to the variability of spindles across cortical regions (*5*, *8*), their laminar dynamics have received much less attention (*31*, *32*). To the best of our knowledge, layer-specific changes in damping of spindle oscillations has not been previously studied. To this end, we compared the incidence and o-Quality of spindles recorded during NREM sleep along 16-channel laminar probes implanted in S1 and M1 (Fig. 3D). We found that in both S1 and M1, the total incidence of spindles and high–o-Quality events in particular were highly layer and region specific (Fig. 3E). The most prominent spindle activity (*F*_5,30_ = 30.2, *P* < 0.0001), with the highest o-Quality (*F*_5,30_ = 10.3, *P* < 0.0001), was found between layers L2/3 deep, L4, and superficial subdivision of L5. This distribution shifted to somewhat deeper electrodes in M1, where the incidence (*F*_1.9,11.4_ = 4.03 GG, *P* < 0.05) and the proportion of high–o-Quality spindles (M1 *F*_5,30_ = 3.65, *P* < 0.05) were higher in L4 and superficial subdivision of L5 (Fig. 3E). This result is consistent with anatomical evidence indicating that thalamo-cortical projections to M1 form most synapses in L5 and L4 and, to a lesser extent, in L2 and L3 (*33*). Thus, our data suggest that not only spindle incidence but also prominently their o-Quality vary as a function of both cortical area and cortical layer.

The question arises whether spindles with different o-Quality may have different generators. To address this, we compared the LFP and current source density (CSD) magnitude in different layers during LFP spindles with different o-Quality detected in S1 (Fig. 3F). LFP or CSD magnitudes were calculated as the 1-s root mean square (RMS) value centered around each spindle’s maximum-envelope LFP cycle. Across mice, the average spindle laminar profile was consistent, with maximal LFP and CSD amplitudes in layers 2/3 and 4, as described above. The LFP signal amplitude then decreased in layer 5 and yet further in layer 6 (fig. S4). A second, smaller CSD signal was observed in the deeper channels in every animal (Fig. 3F). A repeated-measures analysis of variance (ANOVA) on the laminar LFP and CSD RMS values revealed significant effects of layer and o-Quality on the signal magnitude (*P* < 0.001 in each mouse, LFP and CSD). The laminar profile of CSD was, however, only weakly affected by the spindle o-Quality (i.e., significant interactions between layer and o-Quality; *P* < 0.001) but with only small effect sizes (partial η^2^ < 0.1).

Using the layer magnitudes of all unique spindle events, we performed a principal components analysis (PCA) for every mouse separately. LFP and CSD amplitudes across layers were highly correlated: the first principal component in every mouse was the only component with an eigenvector above 1, with explained variances ranging from 71 to 84% for the LFP and 71 to 81% for the CSD. No distinct clusters were discernible in any mouse for either LFP or CSD, further suggesting that one fundamental laminar profile is indeed highly dominant across all spindles, and spindles with different o-Quality have similar generating networks.

### The degree of network synchrony underpins the o-Quality of sleep spindles

**Fig. 4.**
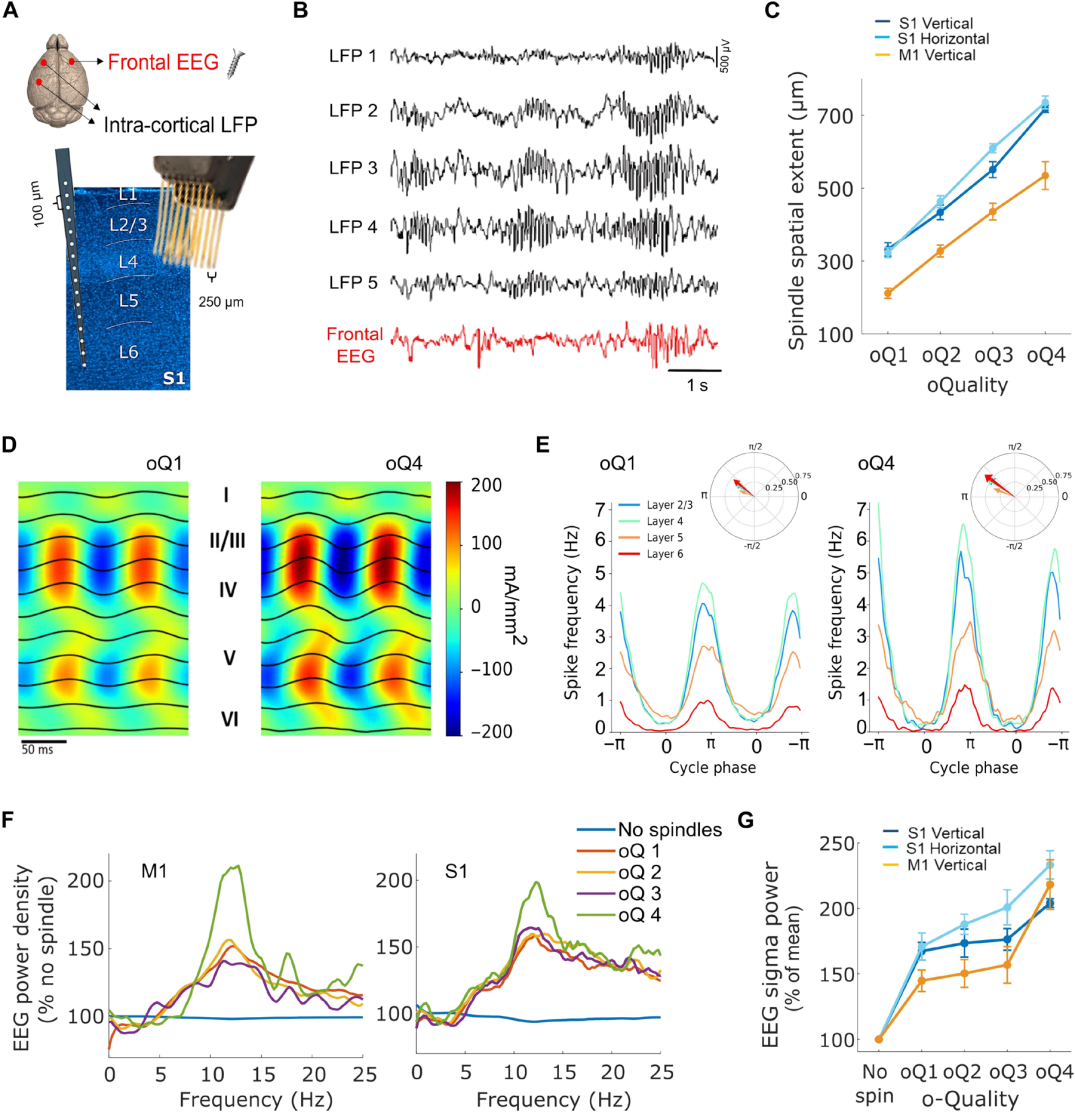
The spindle o-Quality reflects synchrony within local and global cortical networks. (**A**) Diagram showing frontal EEG and LFP (S1 and M1) electrodes. (**B**) Representative S1 LFP and EEG traces with examples of local and global spindle events. (**C**) Spatial extent of LFP spindles as a function of spindle o-Quality (mean, SEM). (**D**) Mean maximum-envelope two-cycle average in a representative animal for oQ1 and oQ4 spindles. LFP traces are superimposed on the spatially smoothed CSD, averaged across all detected spindle events. (**E**) Spiking activity as a function of cycle phase obtained from the Hilbert transform of the LFP in layer 4. Compass plot: mean firing angle and resultant vector length for all spikes within 1 s of spindle midpoint by layer. Rayleigh’s test of circular uniformity confirmed significant phase coupling between MUA and LFP phase in every layer, o-Quality and mouse. (**F**) Mean frontal EEG power spectra during epochs with detected LFP spindle events in M1 (left) and S1 (right) as a percentage of epochs without spindles. (**G**) Mean EEG sigma power in the frontal derivation during epochs with detected spindles as a function of o-Quality of LFP spindles detected in S1 (dark blue, laminar probe; light blue, microwire array) and M1 (orange). Note: figures show mean and, where relevant, SEM across mice. EEG, electroencephalogram; S1, primary sensory cortex; M1, primary motor cortex; LFP, local field potential; SEM, standard error of the mean; CSD, current source density; MUA, multiunit activity. S1 laminar: *n* = 7; S1 micro-array: *n* = 7; M1 laminar = S1 laminar: *n* = 7.

The observation that o-Quality of spindles correlated with their duration (fig. S2B) suggested that this metric may reflect the size of the network involved or the degree of network synchronization during spindling. It is well known from both human and animal studies that spindles can occur in widespread cortical areas, but most spindles are expressed in restricted local areas (*29*, *32*, *34*). In line with this, visual inspection of LFP and multiunit activity (MUA) signals, recorded with multichannel probes that spanned either vertically across cortical layers or horizontally across cortical areas (Fig. 4A), revealed that spindles in S1 and M1 display a significant diversity in their spatial extent (Fig. 4B). In some cases, spindles occurred at the same time in most recording sites, including both the LFP and EEG. In other cases, sometimes just a few seconds later, only a few channels manifested discernible spindling at a given time.

Consistent with our hypothesis, we observed a strong positive association between the spatial extent of LFP spindles and their o-Quality in all cortical regions (S1_vertical_: *F*_3,18_ = 196.89, *P <* 0.001; M1: *F*_1.2,7.2_ = 70.04 GG, *P <* 0.001; S1_horizontal_: *F*_1.1,5.4_ = 327.5 GG, *P <* 0.001), especially in the S1 area for spindles recorded both within and across cortical layers (o-Quality × derivation interaction: *F*_2.5,21.73_ = 2.46 GG, *P <* 0.05) (Fig. 4C). In other words, those events of highest o-Quality were present simultaneously across the largest number of channels, while events of lowest o-Quality were typically restricted to a few recording channels only. In every mouse, around 46 ± 6% (mean ± SEM) of spindles were detected in only one layer and co-occurrence with other layers was a function of layer distance (fig. S5A). While the laminar profile of spindle detections did not change with increasing o-Quality metric, a higher co-occurrence rate was significantly linked to a higher o-Quality metric (one-way ANOVA: *P* < 0.001, fig. S5, B and C). On average, spindles with lowest o-Quality were expressed within a radius ~280 ± 15.4 μm in S1 and ~150 ± 20.6 μm in M1 (i.e., expressed in ~22 to 50% of all LFP channels). As the o-Quality of spindles increased, the spatial extent of their expression gradually increased, until this reached a radius of ~680 ± 25.9 μm in S1 and ~515 ± 35.1 μm in M1. These results suggest that the o-Quality of LFP spindles reflects network synchrony.

We next addressed whether the amplitude of spindles (again measured as the maximum value of the Hilbert transform of the band-pass–filtered signal during individual events) is also related to their spatial distribution. To this end, we replicated the analysis shown in Fig. 4C now for the four amplitude categories, as we did for fig. S3. While there was a strong positive relationship between o-Quality and the spatial extent of sleep spindles, we observed only a weak association between spindle amplitude and their spatial extent for S1, in both a horizontal and vertical axis (S1_vertical_: *F*_3,18_ = 11.56, *P <* 0.01; S1_horizontal_: *F*_1.4,8.5_ = 15.79 GG, *P <* 0.002) (fig. S3F), while for M1, the relationship was not statistically significant (M1: *F*_3,18_ = 2.34, *P* = 0.12). Mixed-model ANOVA revealed significant interaction between category (1 to 4) and metric (o-Quality and amplitude) for all three derivations (S1_vertical_: *F*_1.8,22.2_ = 59.48, *P* < 0.001; S1 _horizontal_: *F*_1.6,16.1_ = 41.38, *P* < 0.001; M1: *F*_1.5,18.3_ = 22.25, *P* < 0.001). This indicates that the spindle o-Quality metric reflects the synchrony of spindle oscillations within local cortical networks better than spindle amplitude.

Although the precise site of origin of individual spindle events is difficult to determine with our (or indeed any) recording approach, we established that the occurrence of spindles in the LFP signals correlated strongly with MUA modulation in the same recording channels (Fig. 4, D and E). Invariably, MUA in all layers was significantly coupled to LFP phase (Rayleigh’s test of circular uniformity: *P* < 0.001 in all layers, o-Quality groups, and mice), and phase-coupled spiking was most prominent in layers 2/3 and 4 in all animals (Fig. 4E). To test the relationship between spindle o-Quality and spiking activity, the mean firing angle and resultant vector length of one LFP channel per layer were averaged within each mouse, and a two-way repeated-measures ANOVA was performed on the pooled averages. This revealed that the mean firing angle was not significantly affected by either layer (*P* = 0.163) or spindle o-Quality (*P* = 0.480), while the resultant vector length increased significantly with o-Quality (*P* < 0.001), but not layer (*P* = 0.238). No significant layer × o-Quality interactions were observed (mean firing angle: *P* = 0.635, resultant vector length: *P* = 0.578), suggesting that the spiking pattern remains largely unaffected across o-Quality levels, save for higher LFP phase-spiking coupling with higher spindle o-Quality. This suggests that the spatial extent of LFP spindle events reflects predominantly locally originating network activity, rather than volume conducted signals, and the o-Quality is a reliable measure of how strongly spiking is modulated during spindle oscillations.

An important question arises as to what extent spindle o-Quality also reflects synchrony within wider cortical networks. To address this question, we made use of simultaneous recordings of both the LFP and the EEG at distant locations (Fig. 4A). First, we assessed the relationship between the occurrence of LFP spindles and the probability of spindling in the distant EEG signal. Consistent with the notion that most spindles are of low o-Quality, we found that 91.7 ± 1.3% (mean ± SEM) of all S1 LFP spindles are not accompanied by EEG spindles. Furthermore, the occurrence of low–o-Quality spindle events in the LFP was associated with a relatively modest increase of EEG spectral power at the spindle frequency range (10 to 15 Hz) during the corresponding epoch, while high–o-Quality LFP spindle events correlated with a prominent spindle frequency peak on the corresponding EEG spectra (Fig. 4, F and G; main effect of o-Quality *F*_1.48,23.74_ = 21.34 GG, *P* < 0.0001, and a significant positive quadratic effect of o-Quality *F*_1,20_ = 5.05, *P* < 0.05 on the EEG power density at 12.5 Hz). Together, these results suggest that the spindle o-Quality reflects the synchrony of wider cortical networks involved in the expression of spindle events.

### Spindle o-Quality correlates with the probability of spindle and slow wave coupling

Our data suggest that spindle o-Quality varies not only between individual events, but also between cortical regions and layers, and correlates with other spindle characteristics, such as their spatial synchronization and their amplitude. This raises the possibility that the spindle o-Quality metric reflects, more generally, the state of the thalamo-cortical network, which changes dynamically as a function of incoming inputs, the state of arousal, and preceding sleep-wake history. Notably, another major sleep oscillation—the slow wave—is also characterized by the occurrence of local and global events, which can travel across the cortex; vary greatly in terms of their amplitude, topography, and spatial extent; and are exquisitely sensitive to the preceding duration of wakefulness and sleep, as well as network excitability (*34*–*36*). To our knowledge, these well-known properties of sleep slow waves have not been directly linked to the oscillatory strength of spindles.

**Fig. 5.**
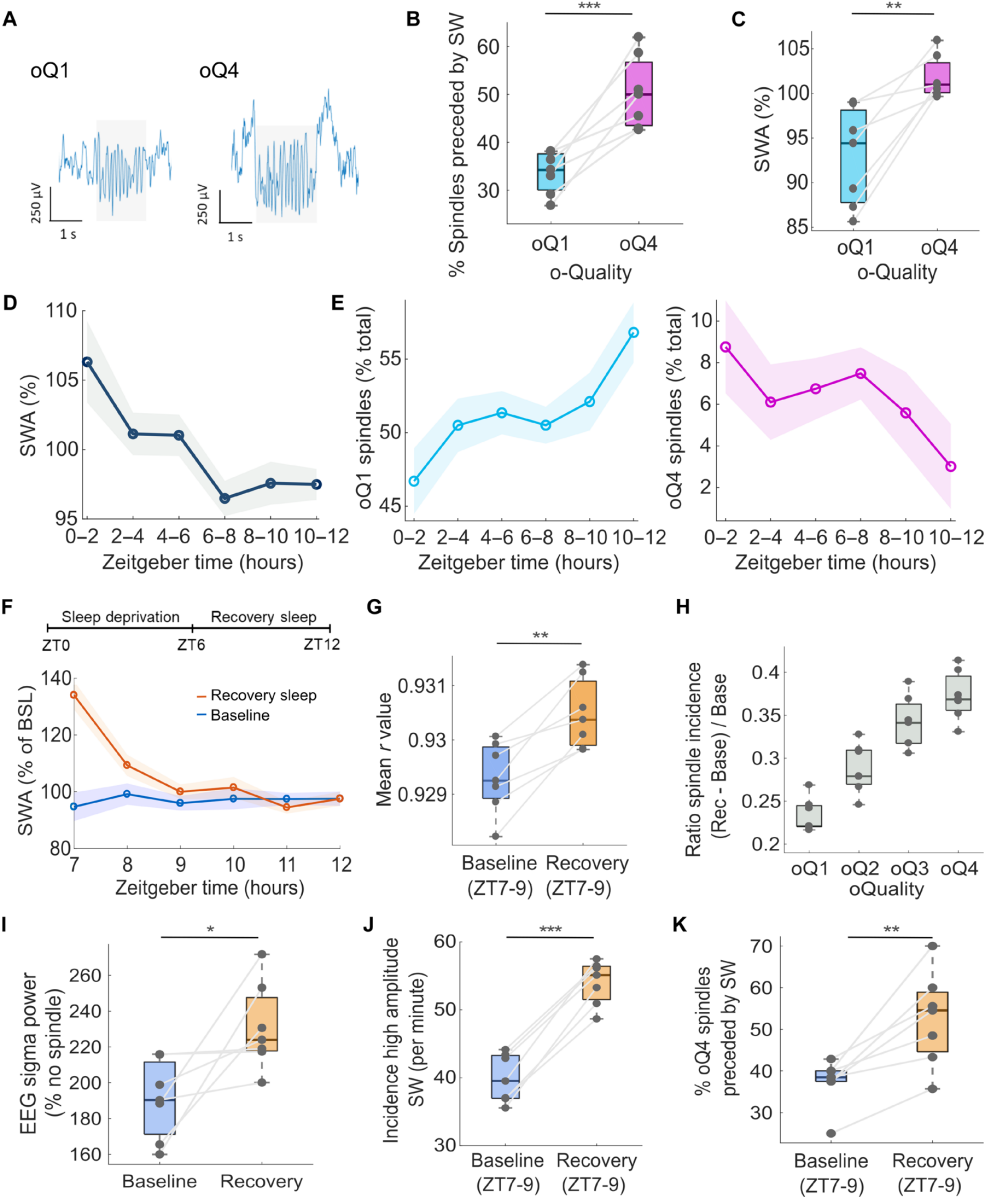
Spindle o-Quality, slow waves, and sleep homeostasis. (**A**) Representative examples of oQ1 and oQ4 spindles in S1. (**B**) Percentage of oQ1 and oQ4 spindles preceded by SW. (**C**) Relative LFP SWA during epochs with oQ1 and oQ4 spindles expressed as percentage of mean NREM SWA. (**D**) Time course of NREM SWA in S1 LFP, expressed as percentage of the 12-hour mean. (**E**) Incidence of oQ1 and oQ4 spindles as percentage of total across the 12-hour light period. (**F**) Time course of NREM SWA in the S1 LFP signal during baseline and after 6-hour SD, expressed as percentage of mean baseline SWA. (**G**) Mean spindle max *r* value during the first 2 hours after SD and corresponding baseline interval. (**H**) Spindle incidence ratio between sleep after SD and baseline as a function of spindle o-Quality. (**I**) EEG 10 to 15 Hz power during epochs with oQ4 LFP spindles during baseline sleep between ZT7-9 and corresponding interval after SD expressed as a percentage of epochs without spindles. (**J**) Incidence of SW during the first 2 hours after SD and corresponding baseline interval. (**K**) Percentage of oQ4 spindles preceded by SW during the first 2 hours after SD and the corresponding baseline interval. LFP, local field potential; SWA, slow wave activity (0.5 to 4 Hz); ZT, Zeitgeber time; SW, slow waves; SD, sleep deprivation. For (D) and (E), dots = mean across mice; shaded areas = SEM. For boxplots: black lines = mean across mice, boxes = SEM, whiskers = 95% confidence intervals, and dots = individual values for each mouse. Analyses were performed on one LFP (S1) channel per mouse that showed the highest spindle density. *P < 0.05, **P < 0.01, ***P < 0.001.

First, we hypothesized that spindle o-Quality is directly related to the probability of coupling between individual slow waves and spindle events. This may be the case given that the spatiotemporal synchrony of spindles is driven by corticothalamic inputs, in which slow waves play an important role (*13*, *37*, *38*), and given that our data suggest that spindle o-Quality reflects local and global network synchrony. To address this hypothesis, we detected individual depth-positive high-amplitude slow waves (0.5 to 4 Hz, see Materials and Methods) in the EEG and LFP signals recorded from layer 5 of S1 using a previously published approach (*39*), and determined the probability of an occurrence of spindle events immediately after a slow wave detection (Fig. 5A).

In general, we found that 3.42 ± 0.4% of all slow waves were followed by a spindle event within 125 ms, and 41.5 ± 2.57% of spindle events were preceded by a locally recorded slow wave, consistent with the notion that only a subset of spindles is nested in slow waves during physiological NREM sleep (*34*, *38*). We found a significant positive association between the probability of slow wave and spindle coupling, and corresponding spindle o-Quality. Specifically, spindle events of higher o-Quality (oQ4) were, in all individual mice, more likely to be preceded by local slow waves than low–o-Quality spindles (oQ1) (*F*_1,6_ = 31.2, *P* < 0.001) (Fig. 5B), and LFP power density in the slow wave frequency range was enhanced during 4-s epochs with high–o-Quality spindle events (*F*_1,6_ = 21.61, *P* < 0.01; Fig. 5C). Notably, the coupling between slow waves and both low–o-Quality (*F*_1,6_ = 199.01, *P* < 0.0001) and high–o-Quality spindles (*F*_1,6_ = 162.6, *P* < 0.0001) was substantially reduced when the time stamps of slow wave occurrence were shifted offline by 700 ms (fig. S6, A and B), which indicates that the slow wave and spindle coupling does not arise by chance.

### Spindle o-Quality reflects network synchrony under increased sleep pressure

Because slow wave activity (SWA), as well as the incidence of high-amplitude slow waves, is sensitive to preceding sleep-wake history (*40*, *41*), we next hypothesized that spindle o-Quality may also reflect homeostatic sleep pressure. Both human and rodent studies have suggested an inverse correlation between EEG SWA and spindle activity dynamics; however, this relationship varies depending on cortical region, specific properties of slow waves and spindles, and the temporal scale used (*6*, *7*, *26*, *42*, *43*). We should point out that little is known about the effects of sleep-wake history on the relationship between SWA and spindles in the somatosensory cortex of mice, and how SD affects oscillatory strength of spindle activity has not been investigated.

Consistent with previous studies, we found that LFP SWA shows a declining time course across the light period (factor time, *F*_5,30_ = 3.63, *P* < 0.01, Fig. 5D), which is the habitual sleep phase in laboratory mice. However, the time course of sleep spindles across the light period varied depending on their o-Quality (interaction o-Quality × time: *F*_5,60_ = 7.77, *P* < 0.0001, Fig. 5E). Specifically, the incidence of low*–*o-Quality (oQ1) spindles increased across the 12-hour light period (ZT 0-12) (*F*_5,30_ = 3.57, *P <* 0.01; linear effect *F*_1,6_ = 7.35, *P* < 0.05), while the incidence of high–o-Quality (oQ4) spindles showed a decreasing time course across this same period (*F*_5,30_ = 8.34, *P <* 0.0001; linear effect *F*_1,6_ = 39.70, *P* < 0.001).

To further address the effects of preceding sleep-wake history on spindle o-Quality, we next performed 6-hour SD, which is a conventional approach to physiologically increase the levels of homeostatic sleep pressure. As expected, LFP SWA increased significantly after SD, which was followed by its gradual decline (interaction condition × time interval *F*_5,60_ = 34.40, *P* < 0.0001, Fig. 5F). Early NREM sleep immediately after SD was also characterized by an increase in the mean o-Quality of sleep spindles relative to baseline sleep (*F*_1,6_ = 27.67, *P* < 0.001; Fig. 5G; increased *r* value = higher o-Quality). Consistently, we also obtained a significant interaction between o-Quality (oQ1 to oQ4) and condition (baseline, recovery) on the incidence of spindles (*F*_3,36_ = 6.31*, P* < 0.001), suggesting that the effects of SD on spindles varied as a function of their o-Quality. This conclusion was supported by the observation of a positive relationship between o-Quality of sleep spindles and the magnitude of their increase after SD (*F*_3,18_ = 37.83, *P* < 0.0001) (Fig. 5H)*.* In addition, we also found a significant interaction between the o-Quality of LFP spindles and sleep condition (baseline, recovery) on the EEG sigma power in the distant EEG signal (*F*_1,6_ = 7.28, *P* < 0.05, Fig. 5I). Specifically, high–o-Quality LFP spindle events resulted in a prominent spindle frequency peak on the corresponding EEG spectra, which was significantly higher during the first 2 hours after SD (ZT7-9) compared to baseline sleep (*F*_1,6_ = 7.8, *P* < 0.05).

Last, we investigated whether the coupling between slow waves and spindles is affected by preceding sleep-wake history. As expected, we found an increased incidence of high-amplitude slow waves during the first 2 hours after SD (ZT7-9) compared to baseline sleep (*F*_1,6_ = 195.7, *P* < 0.0001, Fig. 5J). At the same time, the proportion of high–o-Quality spindles linked with slow waves was 15.2 ± 3.3% higher during the first 2 hours (ZT7-9) of recovery sleep after SD compared to the low sleep pressure condition (ZT7-9) during baseline (*F*_1,6_ = 21.35, *P* < 0.005, Fig. 5K). Notably, this increase in coupling between slow waves and spindles as a function of condition (baseline ZT7-9 versus recovery ZT7-9) was attenuated for low–o-Quality spindles (*F*_1,6_ = 4.54, *P* = 0.09, fig. S5C), and completely abolished when the time stamps of slow wave occurrence were shifted offline by 700 ms (*F*_1,6_ = 1.68, *P* = 0.24, fig. S6D). This indicates that the increase in slow wave and spindle coupling during the first 2 hours after SD does not arise merely by chance but may reflect increased synchronization of the thalamo-cortical network.

Together, these results suggest that spindle o-Quality is a metric that is sensitive to the levels of homeostatic sleep pressure and reflects the state of the thalamo-cortical network under increased sleep pressure. Furthermore, our data provide important insights into the relationship between two major sleep oscillations, which we surmise is determined by the level of network synchronization.

### GluA1-mediated neurotransmission is essential for the large-scale network synchronization of spindles

Our data thus far demonstrate that spindle o-Quality is an informative metric for understanding spatiotemporal synchrony of sleep oscillations. However, the underlying mechanisms linking the network states with oscillatory dynamics remain unclear. To begin addressing the role of spindle o-Quality from a mechanistic angle, we next turned our attention to a recently established mouse model of deficient EEG spindle activity (*26*). These animals, which lack the GluA1 subunit of the AMPA receptor and show impaired synaptic plasticity (*44*), show marked and selective attenuation of spindle power in the frontal EEG during NREM sleep (*26*). The GluA1 subunit plays a key role in a broad range of synaptic functions, and therefore, this mouse model is a promising tool to investigate network mechanisms of local and global spindle propagation. An additional rationale for choosing this model was that recent genome-wide association studies have linked the *GRIA1* gene, which encodes GluA1, with schizophrenia (*45*), and in line with this, GRIA1^−/−^ mice show phenotypes relevant for schizophrenia (*46*). It is well known that EEG spindle activity is markedly reduced in patients with schizophrenia (*47*, *48*) and therefore spindle o-Quality can potentially have a promising and yet untapped clinical relevance in this regard.

**Fig. 6.**
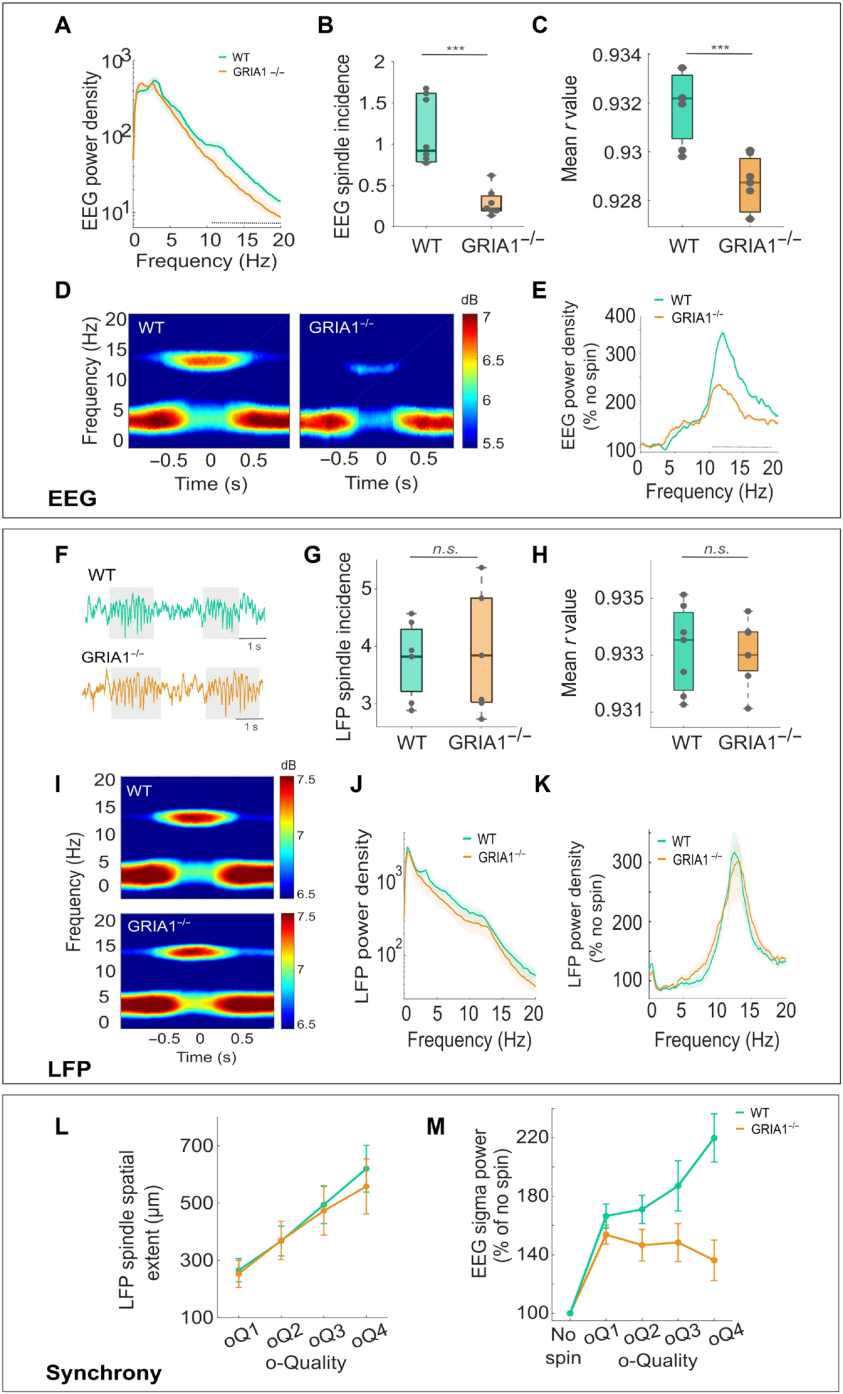
Glutamatergic neurotransmission is essential for large-scale but not local dynamics of spindles. (**A**) Frontal EEG power spectra (mean, SEM) during NREM sleep. (**B**) Frontal EEG spindle incidence per minute of NREM sleep. (**C**) Maximum *r* value for frontal EEG spindles. (**D**) Average spectrograms centered on the midpoint of individual frontal EEG spindles. (**E**) Frontal EEG power spectra during epochs with spindle events, shown as percentage of epochs without spindles (mean, SEM). (**F**) Representative LFP traces (layer 4, S1) with spindles. (**G**) Mean layer 4 S1 spindle incidence per minute of NREM sleep. (**H**) Mean maximum *r* value for LFP spindles detected in layer 4 in S1. (**I**) Spectrograms centered on layer 4 S1 spindle midpoints. (**J**) Average NREM LFP power spectra (layer 4, S1). (**K**) Average LFP power spectra (layer 4, S1) during epochs with spindle events, shown as percentage of epochs without spindles. (**L**) Mean spatial extent of S1 LFP spindles recorded with 16-channel microwire arrays plotted as a function of o-Quality. (**M**) Mean frontal EEG sigma power during epochs with spindles detected in the LFP (layer 4, S1) as a function of their o-Quality. Mean values are shown as percentage of NREM sleep epochs without detected spindles. EEG, electroencephalogram; WT, wild type; SEM, standard error of the mean; LFP, local field potential; S1, primary sensory cortex. Dotted lines: significant differences between genotypes. Boxplots: black lines = mean, boxes = SEM, whiskers = 95% confidence intervals, and dots = individual values. GRIA1^−/−^ (*n* = 7) and WT (*n* = 5) for all panels showing averages across animals. ***P < 0.001; n.s., not significant.

We performed chronic EEG and LFP recordings in freely moving GRIA1^−/−^ mice (*n* = 7) and their WT littermates (*n* = 7) and applied our spindle detection algorithm as described above to the frontal EEG and the LFP recorded in S1. We confirmed (*26*) a marked decrease of EEG spectral power in the spindle frequency range during NREM sleep in GRIA1^−/−^ relative to WT mice (*F*_80,960_ = 8.97, *P* < 0.0001) (Fig. 6A), which, as expected, was associated with a decrease in the total spindle incidence (*t*_7.9_ = 5.21, *P* < 0.001) (Fig. 6B). Furthermore, we also observed that the remaining spindles that were still detectable in the EEG of GRIA1^−/−^ mice were of significantly lower o-Quality than in WTs (*t*_12_ = 4.27, *P* < 0.001) (Fig. 6C; lower *r* value = lower o-Quality) and were associated with an attenuated increase of EEG power in the spindle frequency range (Fig. 6, D and E; genotype × frequency: *F*_80,960_ = 6.47, *P* < 0.0001; effect of Genotype: *F*_1.3,4.1_ = 14.79, *P* < 0.001).

Unexpectedly, visual inspection of LFP signals in S1 revealed the occurrence of well-defined NREM spindle events in S1 in all individual GRIA1^−/−^ mice (Fig. 6F). These events were characterized by a similar incidence (*t*_12_ = 0.32, *P* = 0.71) and o-Quality (*t*_12_ = 0.17, *P* = 0.86) as in WTs (Fig. 6, G and H) and were associated with comparable levels of spectral power in the corresponding LFP signal (Fig. 6, I to K, genotype × frequency: *F*_80,960_ = 0.23, *P* = 0.77; effect of genotype: *F*_1,12_ = 1.35, *P* = 0.27). In addition, in both genotypes, there was a positive relationship between the o-Quality and the spatial extent of LFP spindles (Fig. 6L, genotype × oQ: *F*_1.15,13.86_ = 1.96, *P* = 1.84, effect of o-Quality on spindle spatial extent: *F*_1.15,13.86_ = 91.5, *P* < 0.0001), suggesting that the local synchrony of spindles in S1 is intact in GRIA1^−/−^ mice. However, in the GRIA1^−/−^ mice, the occurrence of S1 LFP spindles, even of high o-Quality, was only weakly associated with any changes in the frontal EEG (Fig. 6M, genotype × oQ*: F*_1.9,22.9_ = 13.89 GG, *P* < 0.0001; KO: *F*_1.19,7.18_ = 1.69 GG, *P* = 0.24; WT: *F*_1.96,11.76_ = 14.98 GG, *P* < 0.001). One interpretation of this finding is that the deletion of the GluA1 AMPA receptor subunit results in a failure of S1 spindles to propagate to distant cortical areas. This finding suggests an important role of GluA1-mediated neurotransmission and synaptic plasticity in the regulation of large-scale network synchronization of sleep spindles.

### The o-Quality of spindles is inversely related with the behavioral responsiveness to auditory stimulation during sleep

Mounting evidence suggests that spindles protect sleep from sensory disruption (*16*–*21*), possibly by reducing the relay of sensory information in the thalamo-cortical network (*49*). Opposing findings have also emerged from studies measuring neuronal activity during natural sleep, which indicate minimal influence of spindle activity on auditory processing during sleep or overall firing rate modulation (*22*). Here, we set out to test the proposed function of spindles in protecting sleep from sensory disruption and explored whether the o-Quality of spindles could be linked to the responsiveness, or lack thereof, to auditory stimulation during sleep.

**Fig. 7.**
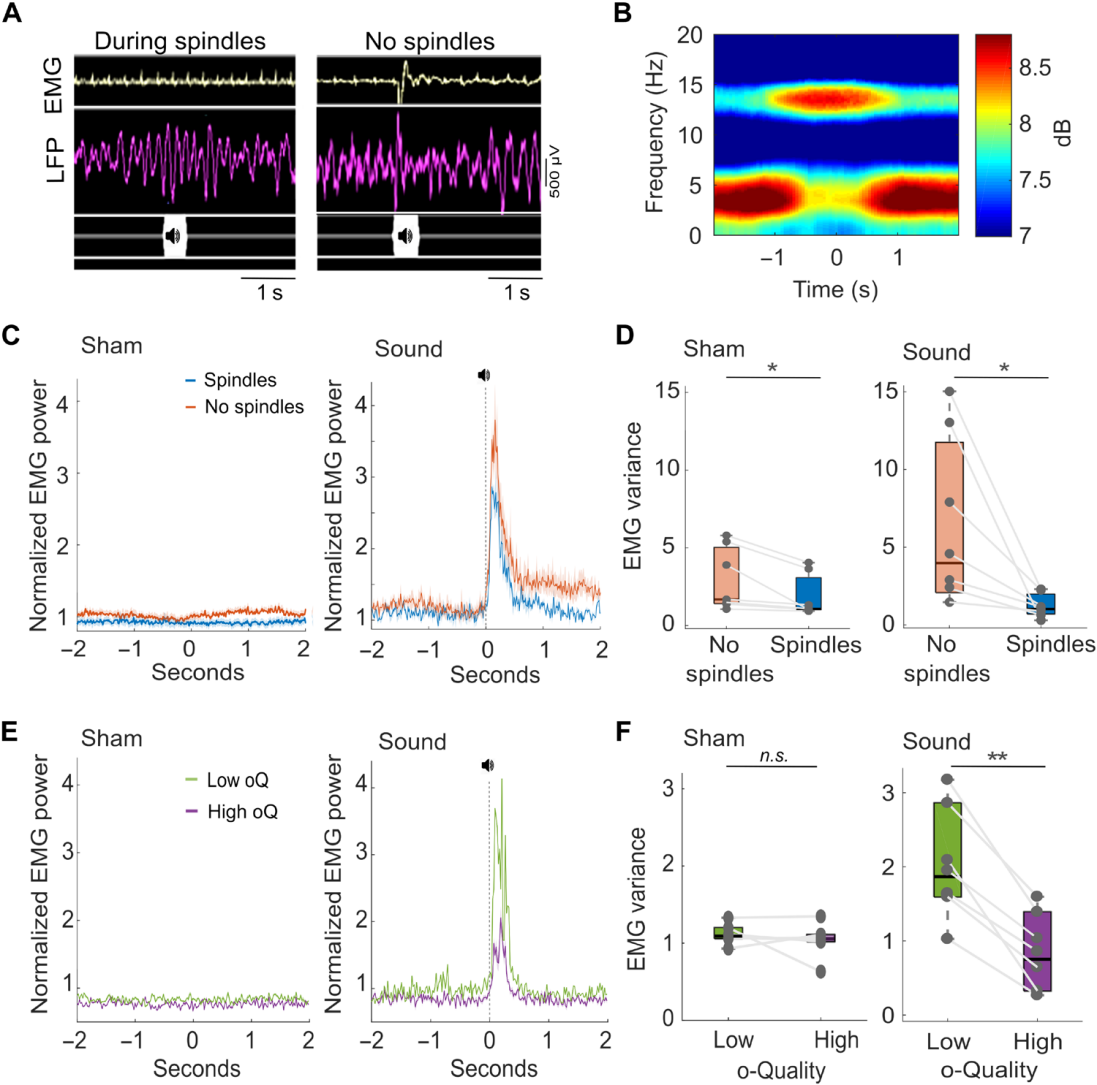
The o-Quality of spindles is inversely related with the behavioral responsiveness to auditory stimulation during sleep. (**A**) Examples of auditory stimulation during (left) and outside (right) spindle events, showing 3-s LFP and EMG segments for one mouse. (**B**) Spectrogram centered around the midpoint of individual spindle events detected in an LFP signal recorded from layer 4 of S1. LFP spectral power represents mean across mice (*n* = 7). Spectrograms are color-coded on a logarithmic scale (dB). (**C**) EMG response to sham stimulation (left) and sound stimulation (right) at time 0, showing stimulations delivered during spindles (*n* = 1400) and stimulations in nonspindle NREM sleep (*n* = 1700). (**D**) EMG variance during the 200-ms period of sham stimulation (left) and auditory stimulation (right) delivered outside or during spindle events. (**E**) EMG response to sham stimulation (left) and sound stimulation (right) at time 0, showing stimulations during spindles of high (oQ4) (*n* = 170) and low (oQ1) (*n* = 780) o-Quality. (**F**) EMG variance during the 200-ms period of auditory stimulation (left) or sham stimulation (right) delivered during spindle events of with high (oQ4) and low (oQ1) o-Quality*.* In (C) to (F), the EMG power (μV^2^) is normalized to the EMG power during NREM epochs with no stimulation. LFP, local field potential; EMG, electromyography. Lines = average across mice, shaded area = SEM. For boxplots: black lines = mean across mice, boxes = SEM, whiskers = 95% confidence intervals, and dots = individual values for each mouse. *P < 0.05, **P < 0.01.

To measure the degree of sensory disconnection, we quantified instantaneous changes in the variance of the electromyography (EMG) signal recorded from the nuchal muscle, in response to sounds played during sleep. To this end, we developed a real-time event-triggered stimulation system, which allowed us to deliver online auditory stimuli during the presence or absence of spindle events detected in S1 (Fig. 7A), the brain area where spindles are most prominent (*18*), and assessed the effects of this stimulation on the EMG response as a readout of sensitivity to the auditory stimulus. Spindles were detected in real time based on the sigma power calculated online from LFP signals recorded from layer 4 of S1 (fig. S7, A and B).

As expected, spectrograms centered at the time stamp of a real-time detection of individual spindles showed a prominent increase in LFP power within the spindle frequency range in S1 (Fig. 7B). The comparative sensitivity (comparative true-positive rate) between detections made with the real-time detector and offline detections with the lowest threshold of the AR model (*r_b_* = 0.92) reached 86.2 ± 2.11%. Auditory stimulation was presented from ZT3.5 to ZT9.5 and consisted of brief (100 ms) pure tones (12 kHz) played at either 70 dB (“sound condition”) or 0 dB (“sham condition”; fig. S7, C and D). The sound and sham conditions were presented on two different days, and the order of presentation was counterbalanced across mice.

We first confirmed that the sound stimulation did not affect the properties of spindle events or the distribution of vigilance states across the 12-hour light period. Specifically, we found that the percentage of time mice spent in NREM (*F*_11,66_ = 0.74, *P* = 0.69), REM (*F*_11,66_ = 1.1, *P* = 0.41), and wake (*F*_11,66_ = 0.87, *P* = 0.60) states, or the number of brief awakenings (*F*_11,66_ = 1.43, *P* = 0.23), did not differ between the sound and the sham conditions (fig. S8, A to C). Similarly, we found that the incidence (*t*_6_ = 0.43, *P* = 0.68), duration (*t*_6_ = 0.16, *P* = 0.88), and o-Quality (*t*_6_ = 0.27, *P* = 0.79) of spindles did not differ during sound relative to sham stimulations (fig. S9, A to D). Similarly, spindle frequency changed by only 0.1 Hz (*t*_6_ = 4.38, *P* < 0.01) between conditions (fig. S9C). LFP power at the sigma frequency range also did not differ with sound relative to the sham stimulation (*z* test = 0.06, *P* = 0.99) (fig. S9E).

Next, we calculated the EMG response to auditory stimulation (i.e., sound versus sham) across spindle conditions (i.e., present versus absent). Considering epochs with sham stimuli (0 dB) only, we found that the EMG variance was significantly lower when spindles were present as compared to trials when spindles did not occur (mean difference = 1.09; *F*_1,6_ = 7.69, *P* < 0.05) (Fig. 7, C and D). This indicates that muscle activity is generally lower during spindle events. Notably, this mean difference in EMG variance was even greater during the “sound stimulation,” when auditory stimulation (70 dB) was delivered at the time of spindles or outside spindle events (mean difference = 5.86, *F*_1,6_ = 7.12, *P* < 0.05), leading to a significant spindle condition (present versus absent) × stimulation (sound versus sham) interaction (*F*_1,2_ = 4.7, *P* < 0.05) (Fig. 7D). These results suggest that, overall, the EMG response is lower at the time of spindle occurrence and the presence of spindles in S1 is related to an attenuated EMG response to auditory stimulation.

Next, we investigated whether the EMG response to auditory stimulation varied in relation to the o-Quality of S1 spindles detected offline with the AR model. We found that the variance of the EMG signal was significantly higher when sounds were played during spindles with low o-Quality relative to spindles with high o-Quality (*F*_1,6_ = 36.11, *P* < 0.01, Fig. 7, E and F). This difference was not present during the sham stimulation condition (*F*_1,6_ = 0.2, *P* = 0.89, Fig. 7, E and F). These results suggest that the o-Quality of spindles is inversely related with the behavioral responsiveness to auditory stimulation during sleep. This is in line with the hypothesis that spindles protect sleep from sensory disruption, but importantly, these findings highlight that not only the presence but also the o-Quality of spindles provides relevant insights into their functional role.

## DISCUSSION

The primary aim of this study was to characterize the heterogeneity of spindles in terms of oscillatory strength and explore the physiological and functional meaning of this variability. It is well known that a wide range of neurophysiological parameters are best described with a lognormal distribution (*50*), and we now demonstrate that this includes the fundamental defining properties of sleep spindles, such as their damping and amplitude. The perspective of relying on quantitative measurements such as the incidence of spindles per topographical location has dominated the field for decades and has been instrumental in understanding their neurophysiological mechanisms and functions (*27*). However, the time is ripe to recognize the importance of not only quantifying spindles but also capturing their variability in terms of oscillatory strength and integrating this aspect into their definition. Doing so will not only advance our comprehension of the neurophysiological mechanisms underlying the dynamics of sleep spindles, but also help to clarify their functional role.

To this end, we propose here the concept of o-Quality, which allows us to measure and parameterize the strength of oscillatory events occurring at the spindle frequency range (10 to 15 Hz). The o-Quality metric is derived by fitting an AR model to short segments of electrophysiological signals and using it to identify and calculate the damping of spindle oscillations. We found that the o-Quality (i) captures a wide range of spindle properties related to their spatiotemporal dynamics, (ii) directly reflects the degree of network synchronization, (iii) correlates with the probability of spindle-slow wave coupling, and (iv) is inversely related to the behavioral responsiveness to auditory stimulation during sleep. These findings, together with the observations that the o-Quality of spindles is sensitive to both manipulations targeting glutamatergic neurotransmission and preceding sleep-wake history, point to the global regulation of synaptic strength as one of its possible neurophysiological substrates.

### The o-Quality of sleep spindles is an emergent property of their spatiotemporal dynamics

The present study supports previous findings, which suggest that characteristics of spindle activity are strongly influenced by the topography of their occurrence (*5*, *8*, *30*). Furthermore, we find that o-Quality of sleep spindles varies substantially between microscopic and mesoscopic regions, and this variability shows distinct topographic gradients. At the EEG level, the spindle o-Quality is higher in anterior cortical areas, while intra-cortically, the o-Quality of spindles was higher in anterior regions of S1 and comparatively lower in M1. However, our results also demonstrate that within a specific cortical region (i.e., S1), one fundamental laminar profile describes all spindles regardless of their o-Quality, suggesting that spindles with different o-Quality have similar generating networks. In addition, we demonstrate that the laminar profile of spindles shows regional variations, which is consistent with previous findings showing that the density of thalamo-cortical projections to different layers varies across cortical areas (*51*). In line with previous studies (*31*, *52*), we found that the incidence and o-Quality of spindles is higher in layers 3 and 4 of S1, while in M1, where thalamo-cortical projections form most synapses in layer 5 (*51*), spindle incidence and o-Quality is highest in the superficial subdivision of layer 5 and layer 4.

### The o-Quality of sleep spindles reflects network synchronization

Overall, our results support the view that spindles are primarily local phenomena (*5*, *6*, *8*, *34*), but spindles can also be observed across large cortical regions (*53*, *54*). Moreover, our results indicate that the o-Quality of sleep spindles reflects the levels of synchronization within and across cortical networks. Specifically, spindles with low o-Quality are typically observable within a few nearby recording sites only, and are transient, while high–o-Quality spindles persist longer and encompass larger cortical regions. The probability of spindles occurring across extensive cortical regions, and thus being evident in the EEG, is influenced by the number of LFP channels exhibiting spindle activity and the o-Quality of this spindle activity. Furthermore, we also showed that there is no discernible difference in laminar profile between local (low o-Quality) and global (high o-Quality) spindles, suggesting that spindles with varying o-Quality share similar generating networks.

Our results show that the vast majority of local LFP spindles remain undetected at the global EEG level. This suggests that studies of spindle dynamics based on “global” EEG recordings should be viewed with caution as these include only a small proportion of high–o-Quality and synchronous events, omitting most local spindles. This is especially relevant as the traditional approach to detect spindle events is based on the amplitude of spindle events detected in the band-pass–filtered signals. Our study unequivocally demonstrates that o-Quality and spindle amplitude show only a weak relationship, and that o-Quality is a more sensitive metric of synchrony within cortical networks.

Our findings imply that the decrease in spindles detected in the EEG in different clinical conditions, may not necessarily indicate dysfunction in spindle-generating regions such as the reticular nucleus of the thalamus, as previously suggested (*48*). Instead, it could potentially signify impairments in the synchronization of spindles across widespread cortical areas. Of course, at present, it is not feasible to obtain intracranial recordings in humans outside of clinical contexts. Nonetheless, our results indicate that high–o-Quality sleep spindles reflect, in general, a more synchronized state of cortical networks, and therefore, it could be a more direct measure of spatiotemporal dynamics and global spread of spindle activity.

To further address the relationship between spindle o-Quality and network states, we tested the hypothesis that oscillatory strength of spindles will correlate with their coupling with other sleep oscillations, such as slow waves. Consistent with this hypothesis, we found that high–o-Quality spindles are more likely to be preceded by high-amplitude LFP slow waves than low–o-Quality spindles. These results are in line with findings suggesting that slow waves are involved in entraining spindle events (*13*, *37*, *38*, *55*). The concurrent increase in SWA and spindle o-Quality after SD also supports the idea that sleep need is associated with a more efficient recruitment of large neuronal populations in network oscillations (*56*). This notion was supported by the observation that the o-Quality is a reliable measure of how strongly neuronal spiking is modulated during spindle oscillations. Together, these results suggest that the o-Quality of spindles reflects synchrony within cortical networks, which is sensitive to the levels of homeostatic sleep need.

Previous studies have shown an inverse correlation between sigma and SWA across the sleep period or during the first hours of recovery sleep after SD (*6*, *42*, *43*). Our results show, however, that the association between SWA and spindles varies based on the spindle o-Quality. Specifically, while the occurrence of low–o-Quality spindles (which show higher overall incidence) shows the typical negative correlation with SWA across the 12-hour light period, high–o-Quality spindles show a positive correlation with SWA. These results raise an interesting possibility that the o-Quality of sleep spindles may be informative about the state of cortical networks in general, beyond being merely a metric specific to sleep spindles only.

### The GluA1 subunit of the AMPA receptor is essential for large-scale but not local dynamics of spindles

To address the underlying neurophysiological mechanisms linking the network states with oscillatory dynamics of sleep spindles, we detected spindle events in transgenic mice deficient of the GluA1 AMPA receptor subunit (*26*). These mice are an important model for investigating the role of synaptic plasticity in behavior and sleep regulation. Surprisingly, we observed that, despite a profound reduction in the incidence and o-Quality of EEG spindles in the frontal cortex, LFP spindles in S1 were preserved in GRIA1^−/−^ mice. Furthermore, despite these S1 spindles showing comparable o-Quality in WT and GRIA1^−/−^ mice, they completely failed to express in distant cortical areas in the animals lacking the GluA1 subunit.

While the exact mechanisms underlying these notable effects remain to be determined, our findings make an important step toward understanding the origin and dynamics of sleep spindles. First, they suggest an important, and hitherto under-investigated link between glutamatergic neurotransmission and the network mechanisms implicated in the generation and propagation of spindles. AMPA and N-methyl-D-aspartate receptors are known to play an important role in the generation of thalamo-cortical oscillations (*57*), but the nuanced role of the GluA1 subunit specifically has not been recognized previously. Crucially, we find that the deletion of this subunit does not affect the capacity to generate spindles or the persistence of spindle activity within local cortical networks. Instead, it primarily affects the large-scale network synchronization of spindle activity, as reflected in spindle events remaining localized and virtually undetectable merely a few millimeters away from the site where they are prominent.

In line with this hypothesis, electron microscopy evidence (*58*) suggests that although thalamo-cortical and corticothalamic synapses in the reticular nucleus of the thalamus express high levels of AMPA receptors, these contain mainly GluA4 and some GluA2/3 subunits. The GluA1 subunit, however, is barely detectable in this brain region. In contrast, GluA1-rich AMPA receptors are expressed in high levels in synapses between thalamo-cortical projecting cells and fast-spiking interneurons in the cortex. It has been suggested that among other mechanisms, these GluA1-rich AMPA receptors could provide rapid activation kinetics capable of recruiting feedforward inhibitory circuits that could propagate spindles across cortical circuits (*59*). There is evidence suggesting that spindle network synchrony is regulated by intracortical connectivity and corticothalamic feedback control (*52*, *53*, *60*). Future studies incorporating targeted ablation of the GluA1 subunit in specific brain regions or cortical layers would be pertinent for advancing our understanding of the mechanisms that govern the widespread synchronization of spindle activity.

These results also have potential clinical implications given the link between GluA1 and neuropsychiatric disorders like schizophrenia. Several studies have suggested that the reduction of EEG spindles in patients with schizophrenia may reflect deficits in the thalamic reticular nucleus in this disease (*47*, *48*). Our results suggest the intriguing possibility that large-scale synchronization deficits, resulting from the disruption of glutamatergic pathways, could alter the expression of spindles at the global EEG level in schizophrenia, even when (at least some) spindle initiation mechanisms are preserved. It is also possible that these spindle disruptions may contribute to the fragmented sleep (*61*) and/or memory deficits (*62*) reported in patients with schizophrenia. Electrophysiological recordings across cortical layers combined with recordings or manipulations of the reticular nucleus of the thalamus would be relevant to further understand the association between GluA1, corticothalamic feedback control, and spindle network synchrony.

Last, given the important role of the GluA1 subunit in the mechanisms of synaptic plasticity, we cannot exclude the possibility that the emergence and propagation of spindle activity during sleep depends on how strong or efficacious the synapses are across the cortex or in thalamo-cortical networks. The functional role of sleep spindles in offline information processing, memory replay, or synaptic renormalization has received considerable attention in the last decades (*59*, *63*). Our data now suggest an intriguing possibility that spindles are, in turn, regulated by the levels of synaptic strength or the capacity to modify synaptic efficacy, possibly in a sleep-dependent manner, which may allow a better understanding of their functional role.

### The o-Quality of spindles is inversely related to the behavioral responsiveness to auditory stimulation during sleep

Evidence suggests that sleep spindles may support the maintenance of sleep by disrupting the transfer of sensory information to the cortex (*16*–*21*). Nevertheless, the neurophysiological mechanisms underlying this effect remain unclear. In addition, conflicting evidence arises in this regard. Some studies suggest minimal modulation of cortical neuron firing rates during spindles (*5*), and other investigations indicate that neuronal responses in the auditory cortex of rats remain largely unchanged regardless of the presence of sleep spindles recorded in that region (*22*).

In our study, we found that motor responses (measured as EMG variance) to auditory stimulation are significantly reduced when stimuli are delivered during spindles compared to NREM sleep in the absence of spindles. Our findings further suggest that not simply the presence, but also the o-Quality of spindles matters, as the magnitude of motor responses to auditory stimulation presented during spindles showed an inverse relationship with the spindle o-Quality. Spindles with high o-Quality are related to a reduced responsiveness to auditory stimulation during sleep, which suggests increased sleep protection.

The potential role of spindles in protecting sleep from environmental disruption has been attributed to the thalamic origin of these oscillations. The thalamus relays sensory information to the cortex and is an important control center that shapes sensation and action, requiring precise inhibitory control, which is largely driven by innervation from structures like the reticular nucleus of the thalamus (*64*). It has been shown that burst firing generated during spindles can quench these sensory inputs (*49*). Specifically, this burst firing reduces the action potential output that thalamo-cortical neurons generate relative to their excitatory input. This has been proposed as one of the mechanisms through which burst firing in thalamo-cortical networks, which gives rise to oscillations like spindles, could reduce the transfer of sensory information to the cortex during sleep (*65*).

In line with these hypotheses, human studies have shown that sensory stimuli fail to generate evoked responses in the cortex and need to have increased intensity to wake participants when stimulation occurs in phase with spindle events detected in the thalamus or cortex (*16*, *18*, *20*, *66*). In addition, the density of EEG spindles during spontaneous sleep positively correlates with the tolerance shown by participants to environmental noise during sleep (*20*). Combined EEG and functional magnetic resonance imaging studies in humans have also shown that pure tones elicit brain responses in the thalamus and primary auditory cortex, which are similar during NREM sleep and wake. These brain responses in the thalamus and the primary auditory cortex are reduced or absent when the sounds are paired with spindles or the down-states of the slow oscillation (*67*, *68*). In addition, mice over-expressing Ca^2+^-dependent small-conductance type 2 potassium (SK2) channels (which have been found to support spindle generation) show enhanced thalamic spindle activity together with decreased responsiveness to noise exposure during sleep (*19*). Our results are in line with these previous findings and further indicate that the spindle o-Quality metric reflects synchrony within the thalamo-cortical network and the o-Quality of spindles affects the responsiveness to auditory stimulation during sleep.

Overall, our study has characterized the dynamics of sleep spindles focusing on their damping, which is one of the primary fundamental properties of any oscillatory activity in nature. The metric we derive, called o-Quality, correlates weakly with the more conventionally used spindle amplitude and provides important insights into our understanding of the neurophysiological and functional relevance of sleep spindles. We provide abundant evidence that the o-Quality of sleep spindles reflects many fundamental properties of spindle activity—from topographical and laminar distribution of spindle events to the synchrony of their generating networks, as well as their coupling with other network oscillations. We demonstrate that most sleep spindles are highly local and therefore not detectable with conventional low-density recording techniques. This makes o-Quality a particularly valuable and informative tool, which allows us to infer the distribution and spatiotemporal dynamics of spindle activity across the brain. Moreover, our results support previous findings suggesting a role of spindles in the protection of sleep from sensory disruption, and further demonstrate that this function is directly related to their o-Quality*.* Shifting attention from reporting how a specific experimental intervention affects the “quantity” of sleep spindles to their o-Quality, in our view, represents a major step forward, which, without doubt, will bring us closer to providing a better mechanistic understanding of brain oscillations in health and disease.

## MATERIALS AND METHODS

### Animals

Experiments were performed in adult male C57BL/6 mice (*n* = 34) and adult male GRIA1^−/−^ (*n* = 7) and littermate WT (*n* = 7) mice [mean age, 16.9 ± 0.5 weeks, and mean weight, 32.5 ± 2.1 g (mean ± SEM) at the time of experiments]. All mice were bred at the Biomedical Sciences Building (University of Oxford, UK). GRIA1^−/−^ mice were generated as previously described (*44*) and maintained on a C57BL/6J × CBA/J background. Heterozygote parents were mated, resulting in ~25% GRIA1^−/−^ mice that lacked both copies of the *GluA1* allele, ~25% WT mice that had both copies of this allele, and ~50% heterozygote mice that were not used here. At the end of all experiments, the genotype of mice was confirmed by genotyping. This was carried out by TransnetYX, USA, using ear notch samples and PCR-mediated amplification methods. During the experiments, mice were individually housed in Plexiglas cages (20.3 × 32 × 35 cm) under a 12-hour light/12-hour dark cycle (lights on at 9 a.m.). Cages were housed in sound-attenuated, electro-magnetic shielded, ventilated Faraday chambers (A Lafayette Instrument Company, USA). Food and water were available ad libitum. Room temperature and relative humidity were maintained at 22° ± 1°C and 60 ± 10%, respectively. Experimental procedures were performed in accordance with the Animal (Scientific Procedures) Act 1986, under a UK Home Office Project License (P828B64BC) and were in accordance with institutional guidelines.

### Surgical procedure and electrode configuration

Surgical procedures were performed under isoflurane anesthesia. All mice (*n* = 48 in total) were implanted with epidural screws to record EEG signals, intracortical probes to record LFPs and MUA, and tungsten wires in the nuchal muscle to record EMG. EEG/EMG mounts were composed of stainless steel screws (shaft diameter, 0.86 mm; InterFocus Ltd., UK) and two single-stranded stainless steel wires, attached to an eight-pin mount connector (8415-SM, Pinnacle Technology Inc., USA) as described previously (*41*). EEG screws were implanted epidurally over frontal [+2 mm anteroposterior (AP), +2 mm mediolateral (ML), relative to bregma], parietal (−0.5 to −1.5 mm AP, 2 mm ML), and/or occipital (−4 mm AP, 2.5 mm ML) cortical regions (fig. S10). A reference screw was implanted over the cerebellum and an anchor screw was implanted contralaterally to the EEG screws to provide stability for the implant. Last, the EMG was recorded from the two stainless steel wires inserted on both sides of the nuchal muscle. All the screws and wires were attached to the skull using dental cement.

LFPs and MUA were recorded across or within cortical layers using two different types of electrode arrays (fig. S10). To record signals across layers of the cortex, mice were implanted with 16-channel laminar probes (NeuroNexus, A1x16-3 mm-100-703, 100 μm spacing), either in the anterior area of the primary somatosensory cortex (S1, *n* = 7 mice, +0.3 mm AP and −3.25 mm ML), or a more posterior area of S1 (*n* = 7 mice, −0.7 mm AP and −3.25 mm ML), or in the primary motor cortex (M1, *n* = 7, 1.1 mm AP and −1.75 mm ML). In a subset of animals (*n* = 7 C57/BL6; *n* = 7 GRIA1^−/−^; *n* = 7 WT littermates), a polyimide-insulated tungsten microwire array (Tucker-Davis Technologies Inc., USA) was implanted into deep layers of S1 (layers 4 and 5), with recording tips positioned approximately equidistant to the cortical surface in the anterior-posterior direction (where well-defined spindles have previously been reported in mice) (*29*, *69*). Microwire arrays consisted of 16 channels with properties as follows: two rows of eight wires, wire diameter 33 μm, electrode spacing 250 μm, row separation L-R: 375 μm, and tip angle 45°. Arrays were customized so that the left row was 250 μm longer. For microwire array recordings, a craniotomy of approximately 1 × 2 mm was made and the midpoint of the craniotomy was located relative to bregma: −1 mm AP and −3.25 mm ML.

### Histological verification of recording sites

To confirm the location of the electrodes (fig. S1), all laminar probes and microwire array wires were coated with DiI fluorescent dye [DiIC_18_ (*3*), Invitrogen], before their implantation. At the end of the experiment, mice were deeply anesthetized, electrolytic microlesions (10 μA, 20 s) were performed at specific sites to be used as landmarks to verify the recording locations (NanoZ, Neuralynx), and mice were transcardially perfused (0.9% saline and 4% paraformaldehyde) as described previously (*36*). Brains were sliced to obtain 50-μm coronal slices, using a Vibratome (Leica VT1000 S, Germany). The brain slices were stained with DAPI (4′,6-diamidino-2-phenylindole), mounted on slides, and imaged with a fluorescence microscope (Olympus Bx51, Japan), using 1.6×, 2.5×, and 5× magnifications. The electrode locations were mapped using the Dil stain and microlesion traces, and the coordinates of the recording sites were identified using a mouse brain atlas (*70*). The depth of the implants was assessed measuring the distance between the cortical surface and the electrical current–induced tissue microlesions (*36*). ImageJ (v1.52a) was used to merge fluorescence images and add scale bars (*71*). All figures were created using Inkscape (v1.0.2, Inkscape Project 2020; https://inkscape.org).

### Signal processing and analysis

Electrophysiological recordings were acquired with an RZ2 High Performance Processor and Synapse software (Tucker-Davis Technologies Inc., Alachua, FL, USA). EEG, EMG, and LFP signals were continuously recorded, concomitantly with extracellular neuronal spike data from the same electrodes used for LFP monitoring (PZ5 NeuroDigitizer preamplifier, TDT, USA).

EEG, LFP, and EMG signals were filtered between 0.1 and 100 Hz and stored at a sampling rate of 305 Hz. The signals were resampled offline at a sampling rate of 256 Hz using custom-made Matlab scripts (The MathWorks Inc., Natick, MA, USA). For subsequent analyses, EEG and LFP power spectra were computed by a fast Fourier transform of 4-s epochs (Hanning window), with a 0.25-Hz resolution Matlab (The MathWorks Inc., Natick, MA, USA).

Extracellular neuronal activity was continuously recorded at a sampling rate of 25 kHz and filtered between 300 Hz and 5 kHz. For spike acquisition, amplitude thresholds were manually set on Synapse on each recording channel (*36*, *41*). This threshold was set at least 2 SDs above noise level. When the recorded voltage crossed this predefined set threshold, 46 samples around the event (0.48 ms before and 1.36 ms after the threshold crossing) were extracted (fig. S11). Spike waveforms were processed using custom-made Matlab scripts. Events with artifactual waveforms were excluded from further analysis.

### Scoring and analysis of vigilance states

Sleep scoring was performed offline and manually (fig. S11). Resampled EEG, LFP, and EMG signals (256 Hz) were transformed into European Data Format (EDF) using open source EEGLAB (Swartz Center for Computational Neuroscience, La Jolla, CA, USA). These signals were visualized in 4-s epochs using the software SleepSign (Kissei Comtec Co., Nagano, Japan). Wake was defined as low-voltage, high-frequency EEG activity accompanied by a high level of EMG activity lasting more than 4 epochs. NREM sleep was defined as signal with high voltage and slow frequency, predominantly characterized by the occurrence of slow waves (0.5 to 4 Hz) and sleep spindles (10 to 15 Hz). REM sleep was defined as low-voltage, high-frequency oscillations, with predominance of theta (6 to 9 Hz) activity in occipital derivations, which was distinguished from waking by the reduced level of EMG activity. Brief awakenings (microarousals) were defined as transient periods of low-voltage, high-frequency oscillations in the EEG and LFP signals accompanied by elevated EMG tone, lasting ≥4 s and ≤16 s (fig. S11). Epochs containing artifacts, resulting from eating, drinking or gross movements, were identified and removed from the analyses. Overall, 11.2 ± 2.9% of wake, 0.6 ± 0.8% of NREM, and 0.8 ± 0.7% of REM epochs contained artifactual EEG and/or LFP signals across all animals.

### Spindle detection

Oscillatory events were detected in all EEG and LFP signals by applying a previously described algorithm (*25*), based on AR modeling of the EEG (see Eqs. 1 and 2). This algorithm models electrophysiological brain signals as a superposition of stochastically driven harmonic oscillators (*f* > 0 Hz) and relaxators (*f* = 0 Hz) with damping and frequency varying in time. For this analysis, filtered (0.1 to 100 Hz) EEG and LFP time-series *x*(*t*) were resampled at 128 Hz and overlapping 1-s segments, shifted by one sampling interval, were modeled with an AR model of order *p =* 8. As such, the AR(8) model uses the weighted sum of the preceding *p* samples to predict the value of the *n*th sample of the time series *x*(*t_n_*)

$$x\left(t_{n}\right)=\sum_{i=1}^{p}a_{i}x\left(t_{n}-i\right)+\varepsilon \left(t_{n}\right)$$ (1)

where *a_i_* denotes the AR coefficients and ε(*t_n_*) indicates the residuals. The model was estimated using the Burg algorithm^167^. These *a_i_* coefficients are related to the frequency *f_k_* = ∅*_k_*(2π∆) and damping coefficient $\gamma_{k}=\frac{1}{t_{k}}=-\Delta^{-1}\;\text{ln}\;r_{k}$ (∆ = *t_n_* − *t*_*n*−1_ denotes the sampling interval) using

$$z^{p}-\sum_{k=1}^{p}a_{k}z^{p-k}=\prod_{k=1}^{p}\left(z-z_{k}\right),\;\;\;z_{k}=r_{k}e^{i{\emptyset \emptyset }_{k}}$$ (2)

Note that *r_k_* is exponentially related to the damping coefficient γ*_k_*, such that a decrease in γ*_k_* is reflected as an increase in *r_k_*. In addition, the order of the AR model (*p*) determines the total number of poles *z_k_* (by *p = 2 m + n*, with *m* oscillators and *n* relaxators), such that our AR(8) model could generate up to four different oscillatory poles *z_k_*. In this way, when the signal is dominated by a rhythmic and stable oscillation with frequency *f_k_*, like a spindle (10 to 15 Hz), this activity will be reflected by a reduction in damping coefficient γ*_k_* and an increase in *r_k_* of the corresponding pole *z_k_* with the frequency *f_k_* (Fig. 2, A to D).

Two types of thresholds were used to detect oscillatory events—named here the upper and the lower threshold. As described previously (*25*), oscillatory events were detected when the damping coefficient γ*_k_* at frequency *f_k_* decreased and, therefore, *r_k_* surpassed a predefined upper threshold *r_b_*. To this end, we have chosen the threshold of 0.92, initially based on the visual inspection of multiple representative recordings in each animal, which revealed that spindle oscillations (10 to 15 Hz activity) were not easily discernible from background activity when *r_b_* was lower than 0.92. To further validate the choice of this threshold and assess to what extent the event detection provides more information than variations in sigma power, surrogate EEG and LFP signals (see the “Surrogate signal generation” section below) were created from the original signals recorded during NREM sleep for every animal and derivation. Next, we calculated the distribution of *r* values for oscillators at frequencies between 10 and 15 Hz for both the original signals and their respective surrogates. We then calculated the ratio between the *r* values of real and surrogate signals (fig. S12). In all cases, the percentage difference between real and surrogate signals was at least 92%, corresponding to an *r_b_* level of 0.92. We acknowledge that this approach is somewhat arbitrary, and the choice of the upper threshold value should be considered operational. This, however, is in line with the key conclusion of our study that spindles are not all-or-none events.

For each event, the start time (*t*_1_) was considered the time point when *r_k_* exceeded *r_b_* = 0.92, while the end time (*t*_2_) was considered the time point when *r_k_* fell below *r_b_* = 0.92. Subsequently, we grouped detected oscillatory events into four groups, named o-Quality (oQ) 1 to 4 (oQ1 to oQ4), corresponding to *r* values between 0.92 and 0.95 (i.e., high-to-low damping), with an additional group including all events above *r* = 0.95 (Fig. 2E). This approach allowed us to either quantify the incidence of oscillatory events within a specific oQ group or investigate the effects of experimental manipulations, brain regions, or cortical layers on the average oQ value across all detected events within the relevant time interval. From a statistical standpoint, the justification for dividing events into oQ categories stems from the nonnormal distribution of *r* values. Treating these *r* values as a continuous metric could potentially obscure relatively large changes in high–o-Quality events due to the high absolute incidence of low–o-Quality spindles.

The lower threshold *r_a_* was used to address transient fluctuations of *r* and to merge or split overlapping, or immediately following, events within a specific frequency. Specifically, consecutive oscillatory detections were considered a single continuous event if *r_k_* stayed above the lower threshold *r_a_* or were considered separate events if *r_k_* fell below *r_a_*. Similar to previous applications (*25*), we set the lower threshold to *r_a_* = 0.90.

The oscillatory events detected by this algorithm in the human sleep EEG correspond to the classically defined EEG frequency bands: e.g., delta (1.5 to 4.5 Hz), alpha (8 to 11.5 Hz), and sigma (11 to 15 Hz) (fig. S2A). Detected oscillatory events that showed a mean frequency *f_k_* between 10 and 15 Hz were defined as putative “spindle events.” Figure 2 illustrates the principle of the algorithm and demonstrates the detection of several spindle events in an 8-s segment of an LFP signal. This spindle detection approach does not require signal filtering in any specific frequency band and does not assume any specific oscillatory waveform.

To enable direct comparison between the o-Quality of spindle events and their amplitude, we band-pass–filtered the signals between 10 and 15 Hz and determined the maximal value of the Hilbert transform for each detected event and then clustered all events into four categories (Amp1 to Amp4) based on the amplitude, where each category included the same number of events as in oQ1 to oQ4 groups.

### Slow wave detection

Slow waves were detected in the EEG and LFP signals following the method presented in (*39*). Specifically, slow waves were detected in the signals after band-pass filtering between 0.5 and 4 Hz, using a phase-neutral (forward-backward) Chebyshev Type II filter (*72*), with stopband edge frequencies at 0.3 to 8 Hz. The parameters of the filter were optimized visually to obtain the maximal resolution of the wave shape, as well as to minimize intrusions of fast frequencies (i.e., spindles). Slow waves were detected as positive deflections in the signal, between two consecutive negative deflections (separated by at least 0.1 s). For our analyses, we selected slow waves with peak amplitudes greater than the median amplitude detected across all slow waves because high-amplitude slow waves accurately reflect homeostatic sleep pressure (*39*) and correspond to well-defined neuronal OFF periods (*41*, *56*). As the amplitude of slow waves is a continuous metric, arguably any choice of a threshold for slow wave detection would be, to some extent, arbitrary; therefore, we would like to emphasize that the approach we used here was not optimized to ensure that all putative slow waves are detected but to focus data analyses on a representative subset of high-amplitude slow waves.

### Surrogate signal generation

We created surrogate data (artificial signals) (fig. S13A) to (i) test the hypothesis that the detected spindle events and observed spindle dynamics were not obtained merely by chance, but rather represent true physiological phenomena, and (ii) substantiate our spindle threshold selection. We considered the approach of surrogate data generation more reliable than relying on a signal without visually detected spindles, which would be subjective. These surrogate signals were created based on an improved version of the iterative amplitude adjusted Fourier transform (IAAFT) algorithm developed by Schreiber and Schmitz (*73*). Specifically, for each mouse and derivation, we calculated a windowed Fourier transform for random 10-min segments of EEG and LFP signals recorded during NREM sleep. The resulting Fourier phases were randomized, and the inverse of the Fourier transform was calculated. Then, the amplitudes of the resulting time series were adapted to match the original amplitude distribution. This was done by rank ordering the resulting time series and replacing the data points with the data point of the original time series with the same rank. This procedure was performed in an iterative manner in order for the surrogate signals to achieve a closer match to the amplitude distribution and the power spectra of the original signals. The comparison of the sigma peak distribution and the infra-slow spectral dynamics of both real and surrogate signals show that the original amplitude distribution of the time series was indeed preserved (fig. S13B) while the endogenous dynamics of spindle activity were not reflected in the surrogates (fig. S13C); i.e., the surrogates did not show the previously reported (*18*, *74*) coordinated 0.02-Hz oscillation of the sleep spindle band.

IAAFT surrogates test the null hypothesis that the data represent a stationary linear Gaussian process observed with a monotone, but potentially nonlinear, measurement function. In our case, we used the 19 LFP (*n* = 19) and EEG (*n* = 19) surrogate signals to assess whether the spindle events detected in the real signal represented true characteristics of an underlying physiological system, or whether they could simply be described by a stationary linear stochastic process (i.e., were obtained by chance). In other words, we used the surrogate signals to assess to what extent the event detection provides more information than measuring variations in sigma power. The selected number of surrogates per signal (*n* = 19) corresponds to a 5% significance level for a one-sided test. In other words, if the observable is larger than the value for all surrogates, the null hypothesis can be rejected (i.e., the detected events are not random).

### CSD and MUA analysis

The analysis on the laminar profile of spindles was done with custom-written Matlab and Python scripts, as well as IBM SPSS Statistics 27. Spindles detected simultaneously in different channels were considered to be co-occurrent (and thus representing the same, unique spindle event) if their centers, as defined from the damping analysis, occurred within 500 ms of each other. The maximum o-Quality metric across channels was then assigned to each unique spindle event. To compare results across mice, every electrode was assigned a cortical layer (2/3, 4, 5, or 6) based on histology. Layer 1 electrodes were omitted from the analysis due to being absent in some mice. The effect of o-Quality on the number of simultaneous detections across layers was determined using a one-way ANOVA for every mouse separately.

Waveform averages as in Fig. 4D were calculated by averaging the unfiltered LFP and the CSD across spindle events, time-locked to the trough of the maximum-envelope cycle in a layer 4–centered channel. The maximum-envelope LFP cycle peaks were aligned across spindle events using interpolation before averaging. For further analysis, the raw LFP signal was band-pass–filtered across the complete recording to a range of 10 to 15 Hz using a fourth-order, zero-phase shift Butterworth filter. Spindle events were extracted from the filtered LFP signal in 5000-ms epochs aligned around the spindle center in the maximum o-Quality channel. The CSD analysis was computed on the band-pass–filtered epoch and smoothed across channels using the cubic interpolation method “interp1d” from the SciPy Python package. The amplitudes of the LFP and CSD signals in each channel were estimated using their respective 1-s RMS from the spindle center. These values were subsequently averaged across all channels within a layer. We used a repeated-measures ANOVA with layers as a within-subject factor and o-Quality as a between-subject factor to evaluate the effect of layer and o-Quality on the laminar profile of spindles, similar to Ujma *et al.* (*32*), and performed this for every mouse. Last, we performed a PCA on the laminar LFP and CSD amplitude profiles of all unique spindles in each mouse separately (fig. S4C).

The instantaneous spindle phase was obtained from the Hilbert transform of the filtered LFP in a layer 4–centered channel. The phase-amplitude coupling of the MUA was calculated by extracting the spike times within 1 s of the spindle center, from one electrode in each layer’s center and assigning to each spike its corresponding spindle phase value (Fig. 4E). Circular statistics (mean angle, resultant vector length, and Rayleigh’s test of uniformity) were performed using the Python “PyCircStat” toolbox. The effect of o-Quality on the laminar profile of the spiking mean angle and resultant vector length was assessed with a two-way repeated-measures ANOVA (with layers as a within-subject factor and o-Quality as a between-subject factor), after averaging the mean angles and vector lengths across spindles for each quality. As the mean firing angles were within a small range (<0.5 radians) and thus nonperiodic, no circular statistics were used for the ANOVAs on the pooled averages.

### Sleep deprivation

To investigate the effect of preceding sleep/wake history and sleep pressure on the characteristics of sleep spindles, slow waves, and spectral parameters, total SD was performed. This was done during the circadian phase when mice are typically asleep and therefore the homeostatic response to sleep loss can be reliably measured (*41*). To achieve SD in an ecologically relevant manner, at light onset, the nesting material was removed from the home cages, and mice were presented with novel objects to induce spontaneous exploratory behavior. This intervention was performed the day following a 24-hour undisturbed (baseline) recording. At the end of 6 hours of SD, all objects were removed, and the nesting material was returned to the cages. The procedure was successful, as mice spent only a minimal percent of the time asleep during SD (1.19 ± 0.42% of 6 hours; *n* = 14).

### Auditory stimulation based on real-time spindle detection

#### Real-time spindle detection

To date, only a few studies have developed real-time spindle detection algorithms (*75*–*78*), and fewer studies have performed real-time acoustic stimulation triggered by spindle detections (*76*). Here, we developed and applied a real-time spindle detection algorithm to deliver auditory stimulation triggered by spontaneous activity in rodent electrophysiological signals using the software “Synapse” (Tucker-Davis Technologies Inc., Alachua, FL, USA) (fig. S7). The delivery of auditory stimuli was timed by the real-time detection of putative spindle events detected in one LFP signal per mouse recorded from S1. Specifically, for each mouse, the recording channel showing the highest incidence of spindles (layer 4) was chosen for real-time detection. The selected LFP signals were first filtered with a high-pass filter at 0.1 Hz and a low-pass filter at 100 Hz, and a second-order parametric filter with center frequency at 12.5 Hz and a fractional bandwidth of 0.4 (octaves) was then applied (to filter the signal between 10 and 15 Hz). Parametric filters are efficient for boosting the signal band of interest and making the attenuation of signals outside the selected band sharper, so their roll-off (i.e., in our case: 9 or 16 Hz) is low (*79*). We then calculated the square of the filtered signal and used an exponential smoothing function, which applies an exponentially decreasing weight to the data as a function of time. A threshold was then set to detect spindles based on the square of the filtered signal. The threshold for real-time detection was set at 4.5 times the mean of the smoothed power signal in line with previous automated detection algorithms (*5*, *69*, *76*).

To restrict the detection of spindles to NREM sleep (i.e., avoiding REM sleep and movement), we set two conditions that had to be met for the algorithm to detect a putative spindle event. First, we calculated the square of the EMG signal, and a threshold was set for each mouse to distinguish between movement and immobility. Second, we applied a second-order parametric filter to the occipital EEG channel to filter the signal in the theta frequency range, which is prominent during REM sleep, and calculated online the square of this filtered signal. A corresponding threshold was set on this filtered signal for each mouse, to identify REM sleep. If either of these two conditions were met (i.e., mobility or high theta power in the occipital derivation), no putative spindle event was detected.

#### Auditory stimulation

Open-field auditory stimulation was performed in the home cages, where mice were single-housed. The home cages consisted of 390 mm by 410 mm by 350 mm electromagnetic shielded and sound-attenuated Faraday chambers (Lafayette Instrument, USA). Sounds were played through magnetic speakers (MF1 Multi-Field Magnetic Speakers, Tucker-Davis Technologies) mounted on the chamber ceilings. Auditory stimuli were designed and triggered with the software Synapse (Tucker-Davis Technologies Inc., Alachua, FL, USA).

A pilot auditory-stimulation session during sleep was performed in three mice to identify sound parameters that generated an evoked response in S1 and an EMG twitch without inducing a global state of arousal. EMG was used as a readout of behavioral responsiveness, as we noticed that it was readily induced by stimulation, and we considered it a more consistent variable as compared to cortical activity that varied markedly, for example, depending on the occurrence of spindle events or slow waves. Fifty different sounds, which ranged in frequency (between 4 and 16 kHz) and intensity (between 60 and 90 dB), were used for this pilot. Pure tones played at 12 kHz, for 100 ms, with an intensity of 70 dB reached the best compromise, and therefore, these parameters were used for the real-time stimulation. In addition, a sham stimulation condition of 12-kHz pure tones played at 0 dB for 100 ms was used as a control. The sound intensity was calibrated using a sound level meter and calibration kit (Grainger, USA).

The sound and sham conditions were presented on two different days, and the order of presentation was counterbalanced across mice. Each day, auditory stimulation was performed for 6 hours during the light period, specifically between ZT3.5 and ZT9.5. Sounds were delivered during (“spindle” condition) or in the absence of spindles (“no-spindle” condition) in a pseudo-random order. A minimum interstimulus interval of 3 s was allowed. Approximately 550 ± 30 stimuli (*n* = 6 mice) were played during the 6 hours of auditory stimulation. Figure 7A shows examples of spindle and no-spindle trials.

Manual sleep scoring and offline automated spindle detection based on AR modeling (*25*) were used to evaluate the performance of the online spindle detection and stimulation algorithm. Overall, 98.3 ± 0.3% of the auditory stimuli were presented during NREM sleep. In addition, the comparative sensitivity (comparative true-positive rate) between detections made with the real-time detector and offline detections with the lowest threshold of the AR model (*r_b_* = 0.92) reached 86.2 ± 2.11%. We calculated the EMG response to auditory stimulation across conditions [real sound versus sham condition presented during (spindle condition) or outside (no-spindle condition) spindle events]. For the spindle condition, only auditory stimuli presented during the occurrence of spindle events confirmed by detection by the AR model were included in the analysis.

### Statistical analyses

Data were analyzed using MATLAB and its Statistics Toolbox (The MathWorks Inc.) and IBM SPSS Statistics (IBM Corp). Linear mixed models ANOVA, factorial repeated-measures ANOVA, repeated-measures ANOVA, *t* tests, and respective nonparametric tests were used as appropriate. To assess differences between specific groups, post hoc tests were performed. The Tukey test was used to compare between groups when equal variances were assumed. The Sidak test was used to do multiple comparisons in cases where equal variances were assumed. Last, the Games-Howell test was used when equal variances were not assumed.

The parametric analyses mentioned above (ANOVA-based, *t* test, and linear mixed models) require dependent variables and residuals to be normally distributed (although they are robust to violations in this assumption when group sizes are equal) (*80*). Shapiro-Wilk normality tests were used to determine whether the data were normally distributed. In cases where the normality assumption was highly violated (and the size of the compared groups was different), either the data were transformed or nonparametric statistics were performed (i.e., Friedman test or Games-Howell post hoc test).

Mixed and multivariate tests require the variances for each combination of the groups to be homogeneous. The Levene statistic was used to test for homogeneity of variance in the different assessed variables (*80*). In cases where the homogeneity of variance assumption was violated, Welch’s *F* test or nonparametric post hoc tests (Games-Howell) were used. In the case of mixed models and repeated measures, the variances of the differences between groups of the within-subject factor (across the between-subjects factor) are required to be homogeneous (i.e., sphericity assumption) (*80*). Mauchley’s test of sphericity was used to assess whether the population variances of all possible different variable combinations were equal. In cases where the sphericity assumption was violated, the Greenhouse-Geisser (referred to as “GG”) correction was applied. This method corrects for the inflation in the *F* value caused by lack of sphericity (unequal population variance at all variable levels) by multiplying the GG estimate by the degrees of freedom used to calculate the *F* value (*80*). In tables and figures, significance levels are indicated with black asterisks as follows: *P < 0.05, **P < 0.01, ***P < 0.001.
