# Supplementary material for: Oscillatory-Quality of sleep spindles links brain state with sleep regulation and function
Source: Sci Adv. 2024 Sep 6;10(36):eadn6247. doi: 10.1126/sciadv.adn6247 (PMC11378912; doi:10.1126/sciadv.adn6247)
Supplement: Supplementary file 1 — Figs. S1 to S13 [file sciadv.adn6247_sm.pdf]

Science Advances
AAAS

Supplementary Materials for

Oscillatory-Quality of sleep spindles links brain state with sleep regulation and function

Cristina Blanco-Duque *et al.*

Corresponding author: Cristina Blanco-Duque, cblanco@mit.edu; Vladyslav V. Vyazovskiy, vladyslav.vyazovskiy@dpag.ox.ac.uk

Sci. Adv. **10**, eadn6247 (2024)
DOI: 10.1126/sciadv.adn6247

This PDF file includes:

Figs. S1 to S13

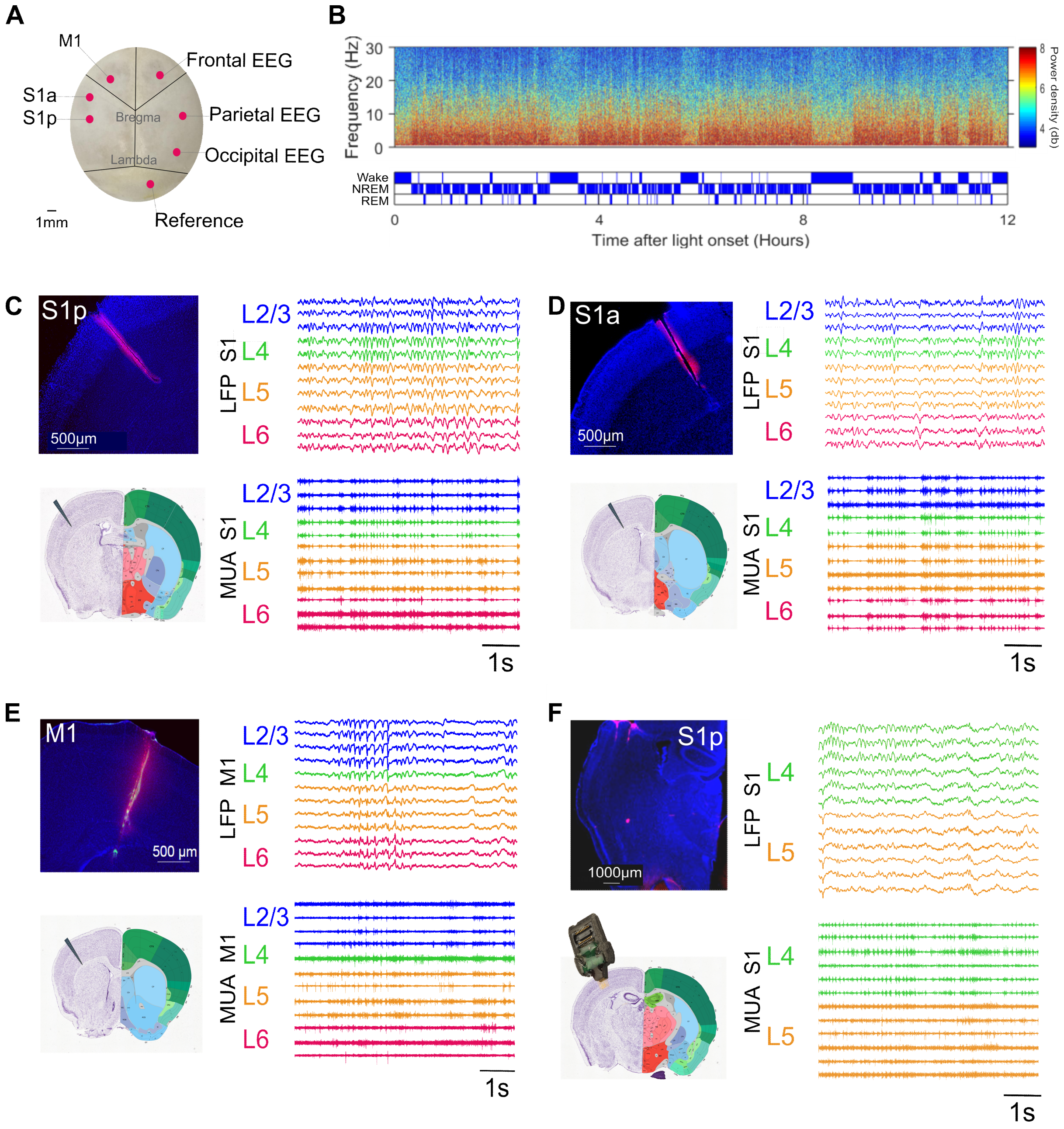

Supplementary Fig. S1 | Recording sites and recordings of cortical activity during wake and sleep. (A) Locations where the EEG screws (frontal, parietal or occipital), LFP laminar probe or micro-wire arrays (M1, S1 anterior, S1 posterior) and reference screw (cerebellum) were implanted. **(B)** Spectrogram (top) and respective hypnogram (bottom) for one mouse during an undisturbed 12-hour light period. The spectrogram is colour-coded on a logarithmic scale. **(C,D,E,F)** Twenty-one mice were implanted with either: a 16-channel laminar probe in S1 anterior cortex (n=7) (C), S1 posterior cortex (n=7) (D), M1 cortex (n=7) (E), or a 16-channel micro-wire array in deep layers of S1 (n=7) (F). In (C,D,E,F), the location of the electrodes across or within S1 and M1 cortical layers was verified with histology of electrolytic microlesions and Dil stain electrode traces (magenta) on brain slices stained with DAPI (blue). The recording coordinates for each implant type were identified using the Allen mouse brain atlas. Representative 7-s segments of LFP and MUA signals (recorded during NREM) sleep across electrodes is shown for the different implants. (*Note:* EEG: electroencephalogram. LFP: local field potential. M1: primary motor cortex. S1: primary sensory cortex. MUA: multi-unit activity).

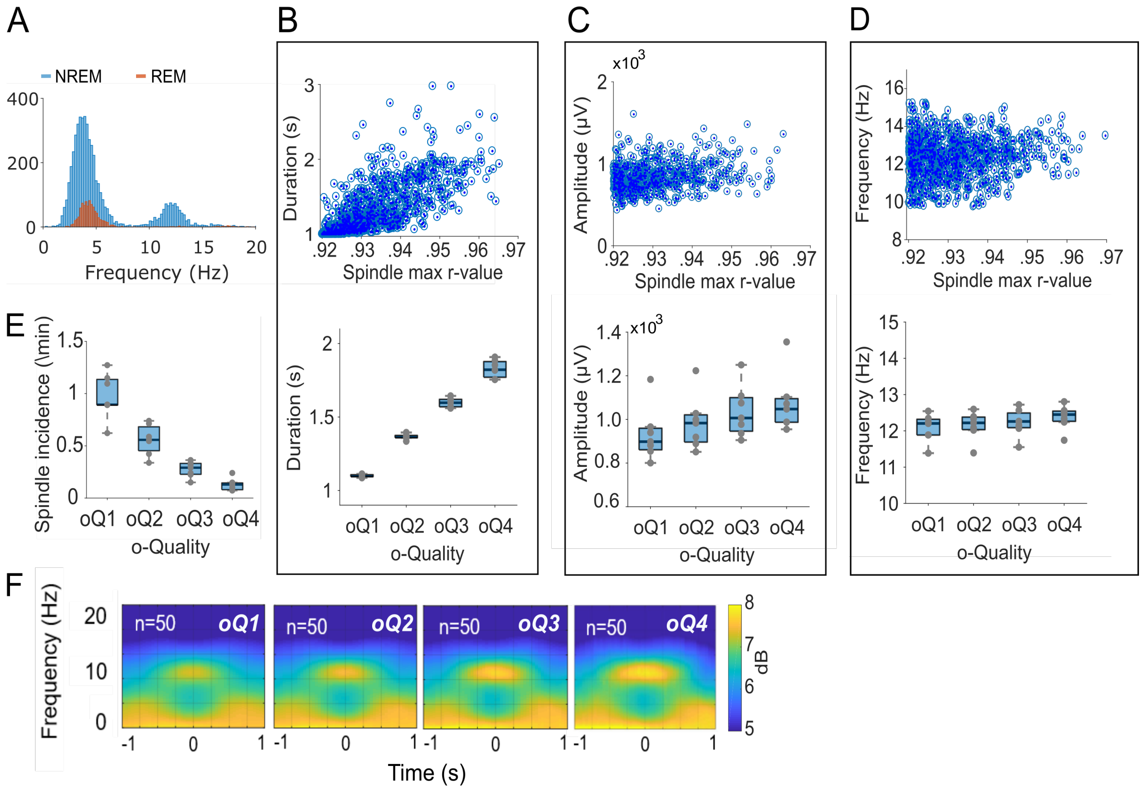

Supplementary Fig. S2 | Properties of spindles as a function of their *o-Quality*. (A) Frequency distribution of all the oscillatory events detected by the AR-model during NREM (blue) and REM (red) sleep from electrodes located in layer 4 of S1 (the cortical layer with highest spindle density). **(B-D)** *Top:* Representative examples of the distribution of spindle duration, amplitude, and frequency as a function of the maximum *r*-value for each detected spindle in one mouse. *Bottom:* Mean duration, amplitude, and frequency of spindles with different *o-Quality*. **(E)** Spindle incidence per minute as a function of their *oQuality*. **(F)** Average spectrograms of 2-s segments of LFP signals recorded from electrodes located in layer 4 of S1. Spectrograms are aligned to the midpoint of each spindle event and averaged across 50 random spindles per o-Quality level and across mice. (Note: For figures B-D: n=7 mice. For boxplots: black lines= mean across mice, boxes= SEM, whiskers= 95% confidence intervals, dots= individual values for each mouse. AR: autoregressive. LFP: local field potential. S1: primary somatosensory cortex. SEM: standard error of the mean).

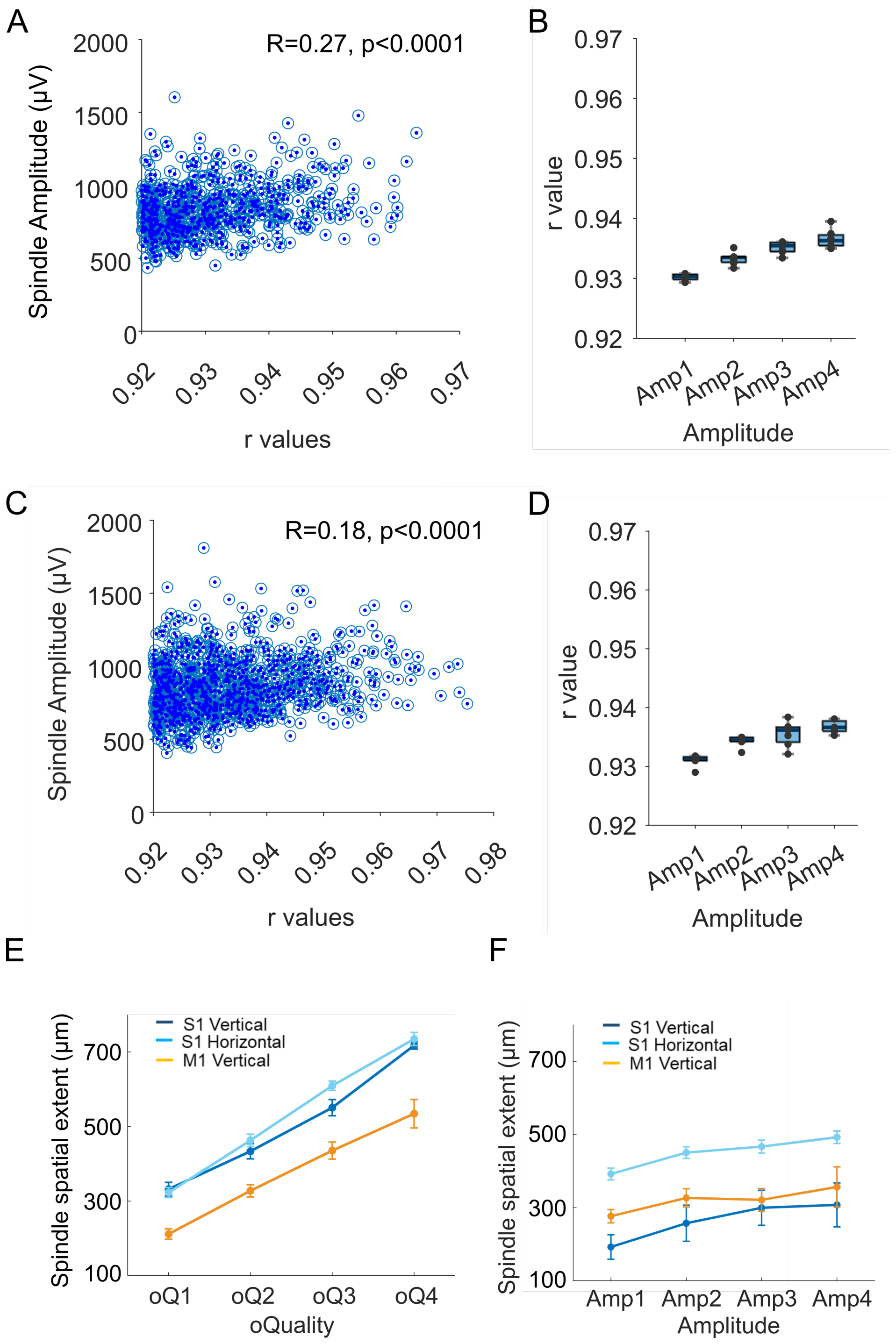

Supplementary Fig. S3 | Spindle amplitude shows weak correlation with the *o-Quality* and the spatial synchronization of spindles. **(A)** Representative example of the distribution of spindle amplitude as a function of the maximum *r*-value for each spindle detected with the AR-model in one mouse. **(B)** Mean *r*-value across mice (n=7) of spindles detected with the AR-model and grouped into four amplitude categories (*Amp1*=lowest amplitude and *Amp4*=highest amplitude). **(C)** Representative example of the distribution of spindle amplitude as a function of the maximum *r*-value for each spindle detected in real-time with a traditional sigma amplitude-based algorithm in one mouse (see Materials and Methods, *Real-time spindle detection*). **(D)** Mean *r*-value across mice (n=7) of spindles detected in real-time with a traditional sigma amplitude-based algorithm, and grouped into four amplitude categories where

Amp1 represents events of low amplitude and *Amp4* represents events of high amplitude. **(E)** Mean spatial extent of LFP spindles recorded from both laminar probes (in S1–dark blue and M1-orange) and micro-wire arrays (S1–light blue), as a function of spindle *o-Quality* (same as Fig. 4C, included for comparison with panel F). **(F)** Mean spatial extent of LFP spindles recorded from both laminar probes (S1–dark blue, M1-orange) and micro-wire arrays (S1–light blue), as a function of spindle amplitude (*Amp1*= lowest amplitude and *Amp4*= highest amplitude). (*Note*: In B and D, boxplots: black lines= mean across mice, boxes= SEM, whiskers= 95% confidence intervals, dots= individual values for each mouse. Figures E and F show mean, SEM across mice and the spatial extent of a spindle event is calculated from the number of LFP electrodes involved in that specific event (as in Fig.4). AR: autoregressive. LFP: local field potential. S1: primary somatosensory cortex. M1: primary motor cortex. SEM: standard error of the mean).

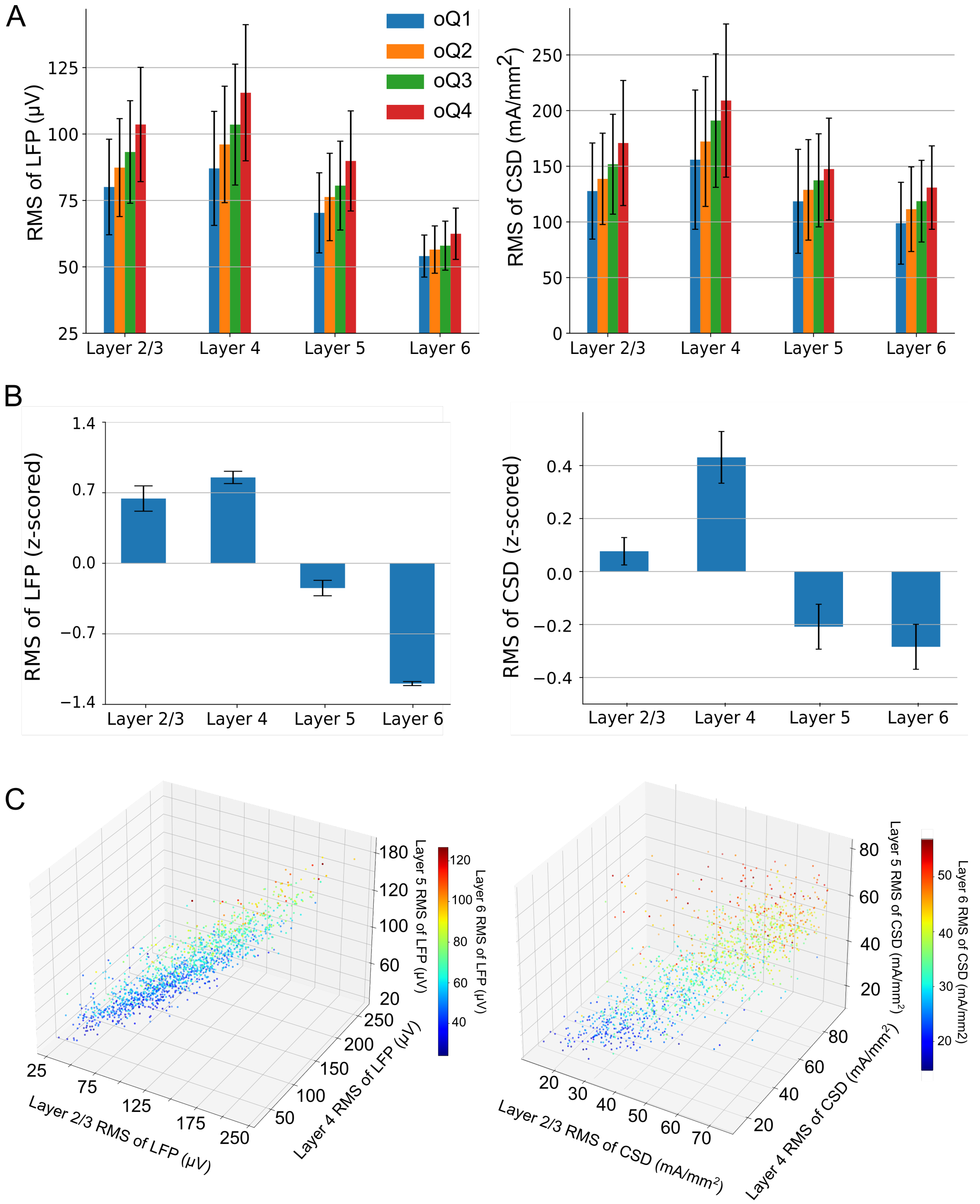

Supplementary Fig. S4 | RMS and CSD profiles of spindles across layers. (A) Laminar profile of LFP (left) and CSD (right) in an example mouse, for each spindle quality. The laminar profile is represented by the average 1-s root mean square (RMS) value from the spindle centre, averaged across channels within each layer. Error bars represent the standard deviation across spindles. **(B)** LFP (left) and CSD (right) laminar profile of all spindles, grand average across mice (n=7). For each mouse, RMS values are z-scored across all cortical channels, and subsequently averaged across channels within each layer and across spindles. Error bars indicate the standard error of the mean. **(C)** 3-D scatterplots showing LFP (left) and CSD (right) RMS for each layer in an example mouse. Each point represents one spindle, with layers 2/3, 4 and 5 plotted onto the x-axis, y-axis and z-axis respectively, and layer 6 represented by the colour scale. Strong collinearity across layers can be observed for both LFP and CSD. (*Note:* LFP: local field potential. CSD: current source density).

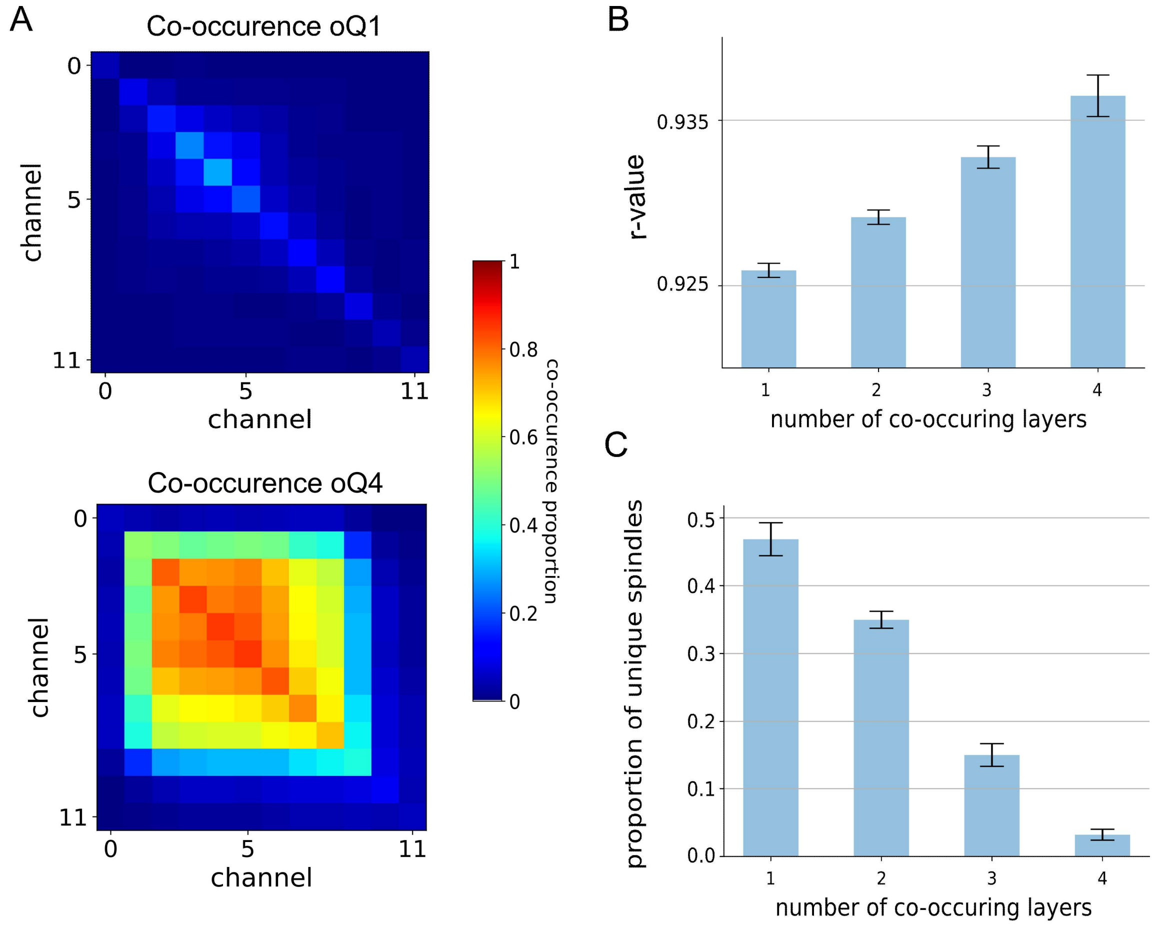

Supplementary Fig. S5 | Co-occurrence of spindles across cortical layers (A) Co-occurrence matrix in an example mouse for spindles of quality one (top) and quality four (bottom). Colour scale indicates the proportion of spindles from each quality that were detected in each channel pair simultaneously. **(B)** Mean quality metric of spindles (r-values) co-occurring in different layers simultaneously in one example mouse. Error bars indicate the standard deviation. **(C)** Group mean across mice of proportion of spindles co-occurring in different layers (n=7). Error bars indicate standard error of the mean (SEM).

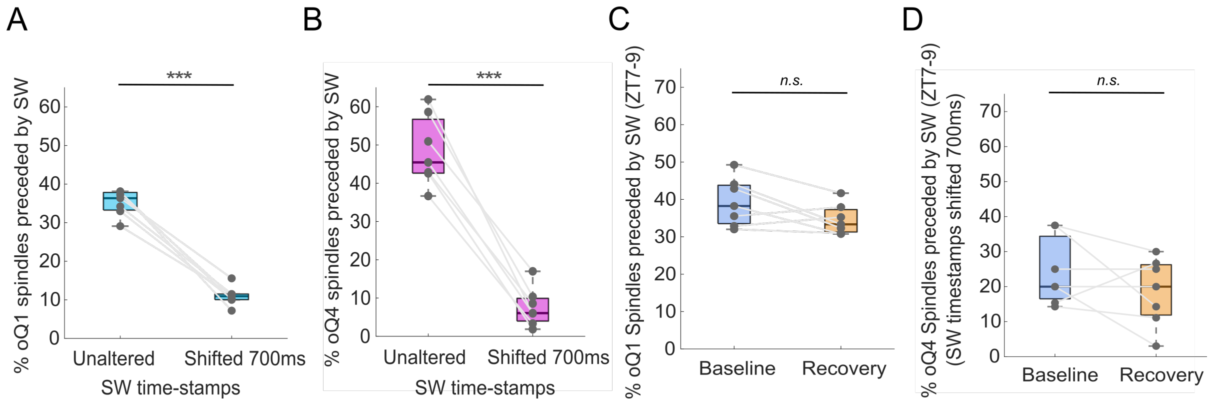

Supplementary Fig. S6 | Coupling of spindles and SW after shifting the timestamps of SW. (A) Percent of low *o-Quality* (oQ1) spindles preceded by SW when SW timestamps (i.e. the start time of automatically detected individual depth-positive high-amplitude SW, see methods '*Slow wave detection*') were unaltered or shifted forward by 700ms. **(B)** Percent of high *o-Quality* spindles (oQ4) preceded by SW when SW time-stamps were unaltered or shifted by 700ms. **(C)** Percent of low *o-Quality* (oQ1) spindles preceded by SW during the first two hours of sleep recovery (ZT7-ZT9) after 6 hours of sleep deprivation (orange) and corresponding baseline sleep time-period (blue). **(D)** Percent of high *o-Quality* (oQ4) spindles preceded by SW (with timestamps shifted by 700ms) during the first two hours of sleep recovery (ZT7-ZT9) after 6 hours of sleep deprivation (orange) and corresponding baseline sleep time-period (blue). (*Note:* For boxplots: black lines= mean across mice, boxes= SEM, whiskers= 95% confidence intervals, dots= individual values for each mouse. SW: slow waves. ZT: zeitgeber time. SEM: standard error of the mean).

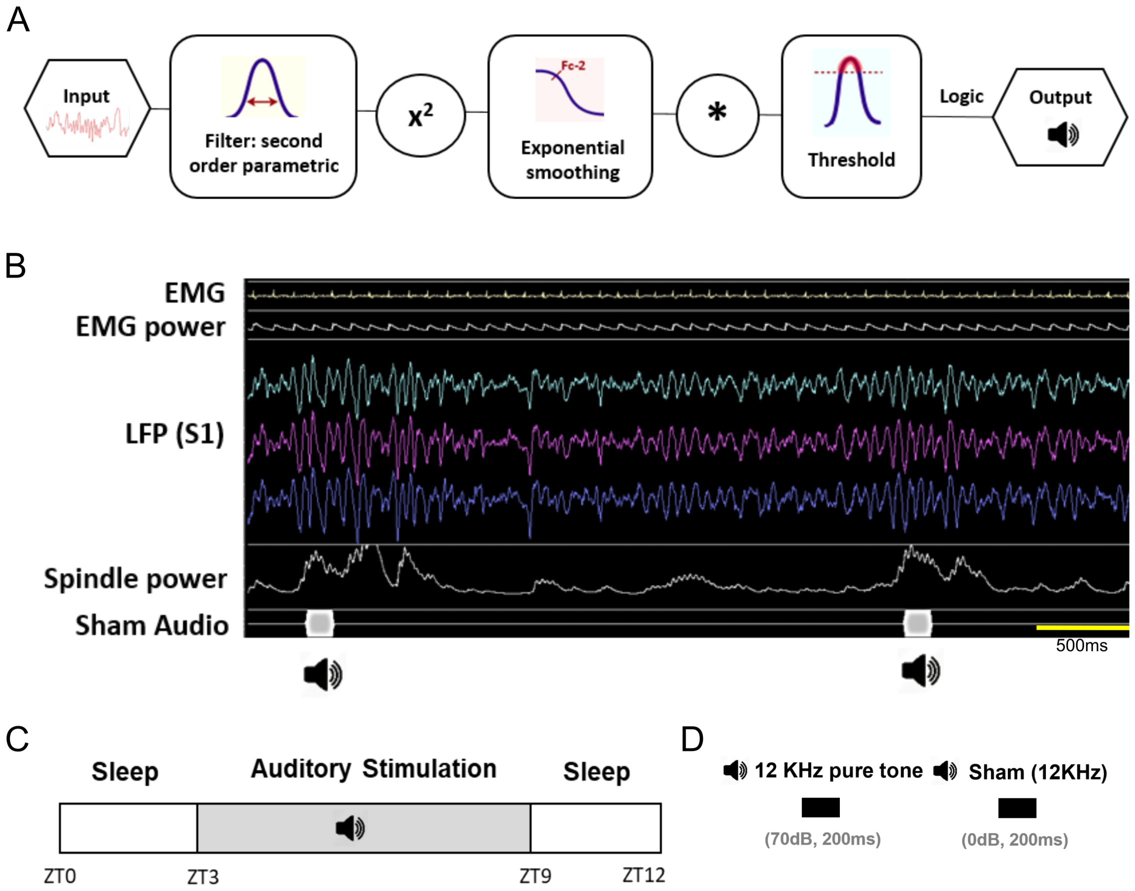

Supplementary Fig. S7 | Principles and schematic of the auditory closed-loop stimulation paradigm. (A) Processing steps followed by the system to detect spindles (10-15Hz) in real time and deliver sounds times-based on these detections. **(B)** Examples of real-time spindle detection in a sham (0dB sound) condition. Spindles were detected when the power of S1 LFP signals increased and reached a predefined threshold. Sounds were delivered only if the EMG and theta power in the occipital EEG were low. **(C)** Schematic of the structure of the auditory-stimulation paradigm performed using the closed-loop system, across 12h light periods (white bar). Zeitgeber Time (ZT) indicates the time from light onset (lights on at 9am; lights off at 9pm). The shaded boxes indicate the times during which the auditory stimulation paradigms were carried out. **(D)** Auditory stimuli consisted of 12kHz pure tones presented at 70 dB or 0 dB (sham) for 200ms. (*Note:* S1: primary somatosensory cortex. LFP: local field potential. EMG: electromyography).

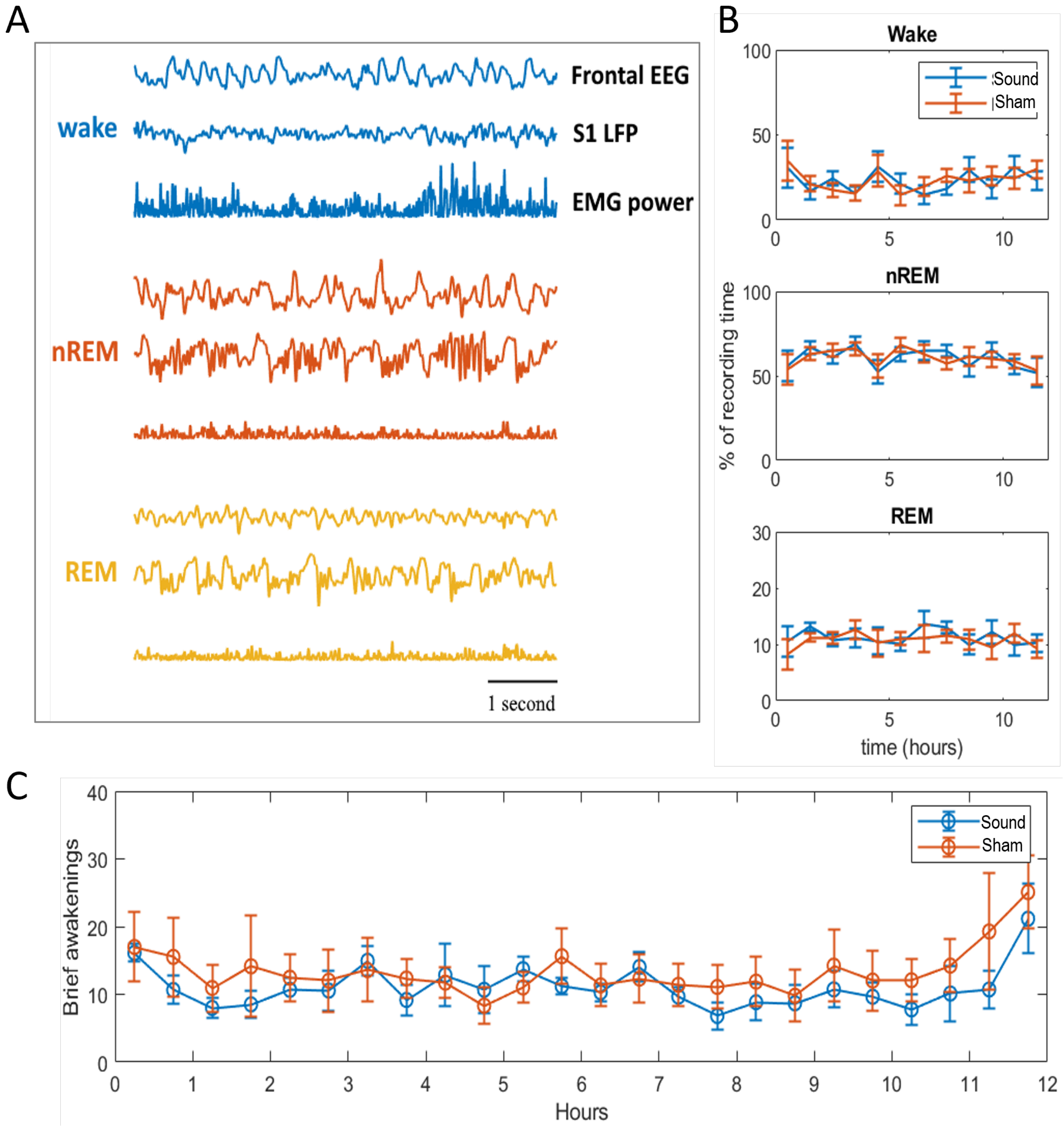

Supplementary Fig. S8 | Auditory stimulation does not affect vigilance states. (A) Representative frontal EEG, S1 LFP, and EMG power traces during wake, NREM, and REM sleep in one mouse. **(B)** Time course of vigilance states over the 12h recording period, in 1h intervals for real stimulation and mock. The amount of each state is represented as a percentage of the total recording time. Mean ± SEM (n=6 mice). **(C)** Brief awakenings during the 12h recording period, shown as number/hr of NREM sleep. (*Note*: EEG: electroencephalography. S1: primary somatosensory cortex. LFP: local field potential. EMG: electromyography. SEM: standard error of the mean).

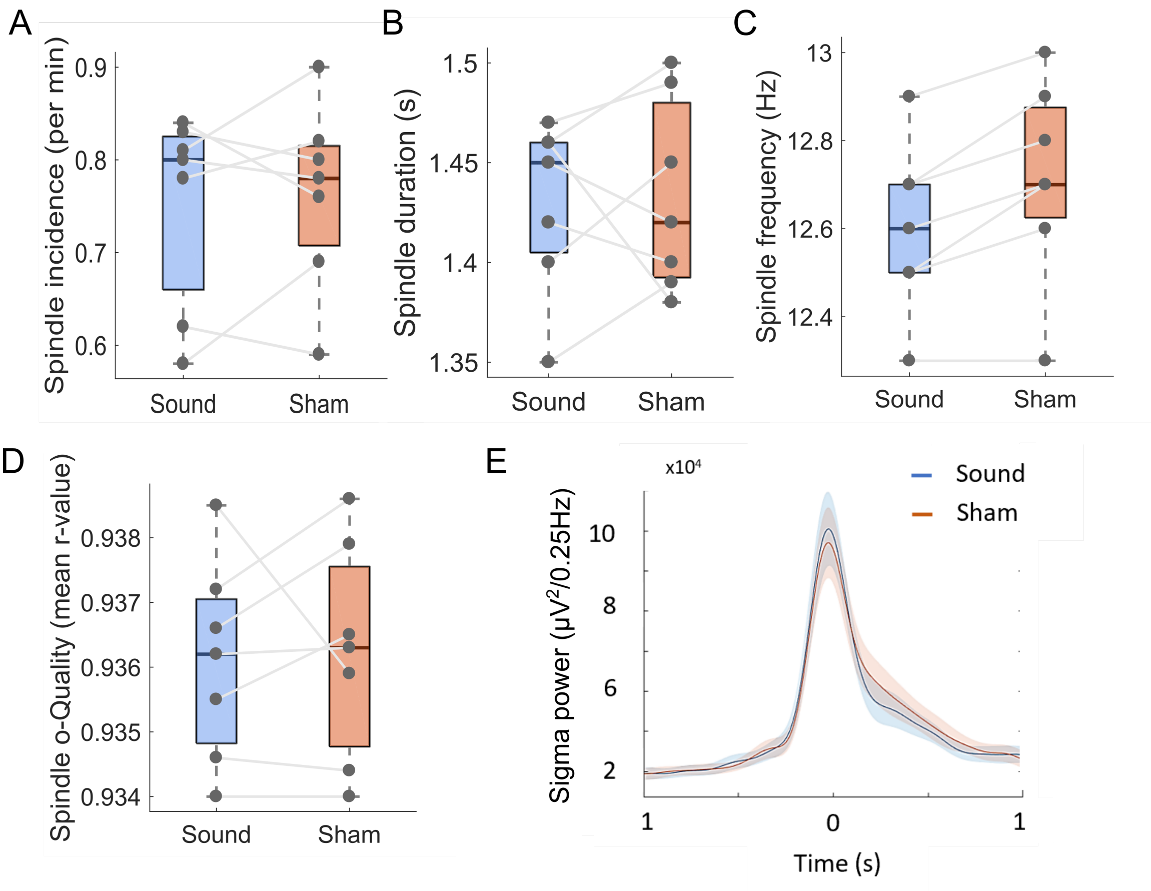

Supplementary Fig. S9 | Spindle metrics are not affected by pure tones. Mean spindle incidence **(A),** duration **(B),** frequency **(C),** and *o-Quality* (higher r-value = higher *o-Quality*) **(D)**, across all mice (n=7) for spindles coincident with auditory stimulation and spindles coincident with sham stimulation. **(E)** Mean ± SEM sigma power time course, where sound stimulation or sham stimulation occurs at time 0s. (*Note:* Lines= average across mice, shaded area=SEM. (*Note:* SEM: standard error of the mean).

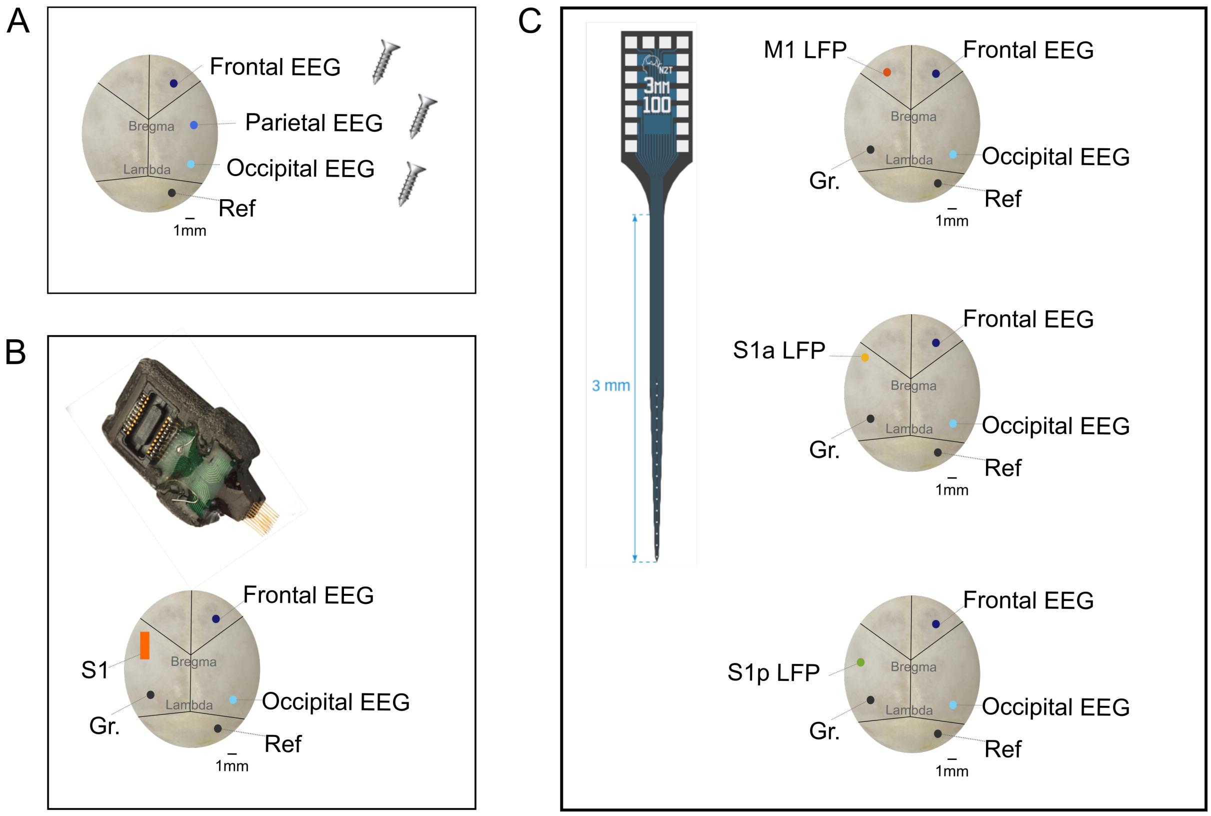

Supplementary Fig. S10 | Electrode placement configurations. (A-C) Locations where the EEG screws, LFP laminar probe or LFP micro-wire array, reference screw (Ref) and ground screw (Gr) were implanted. **(A)** A total of n=6 C57Bl/6 mice were implanted with EEG screws epidurally above the frontal, parietal and occipital cortices. **(B)** A total of n=21 mice (n=7 C57/BL6; n=7 GRIA1-/-; n=7 WT littermates) were implanted with frontal and occipital EEG screws plus a polyimide-insulated tungsten microwire array into deep layers of S1 (layers 4-5). **(C)** A total of n=21 C57Bl/6 mice were implanted with frontal and occipital EEG screws plus a 16-channel laminar probe into the primary motor cortex (M1, n=7), the anterior part of the primary somatosensory cortex (S1a, n=7) and the posterior part of the primary somatosensory cortex (S1p, n=7). (*Note:* EEG: electroencephalography. LFP: local field potential. WT: wild-type. M1: primary motor cortex. S1: primary somatosensory cortex.).

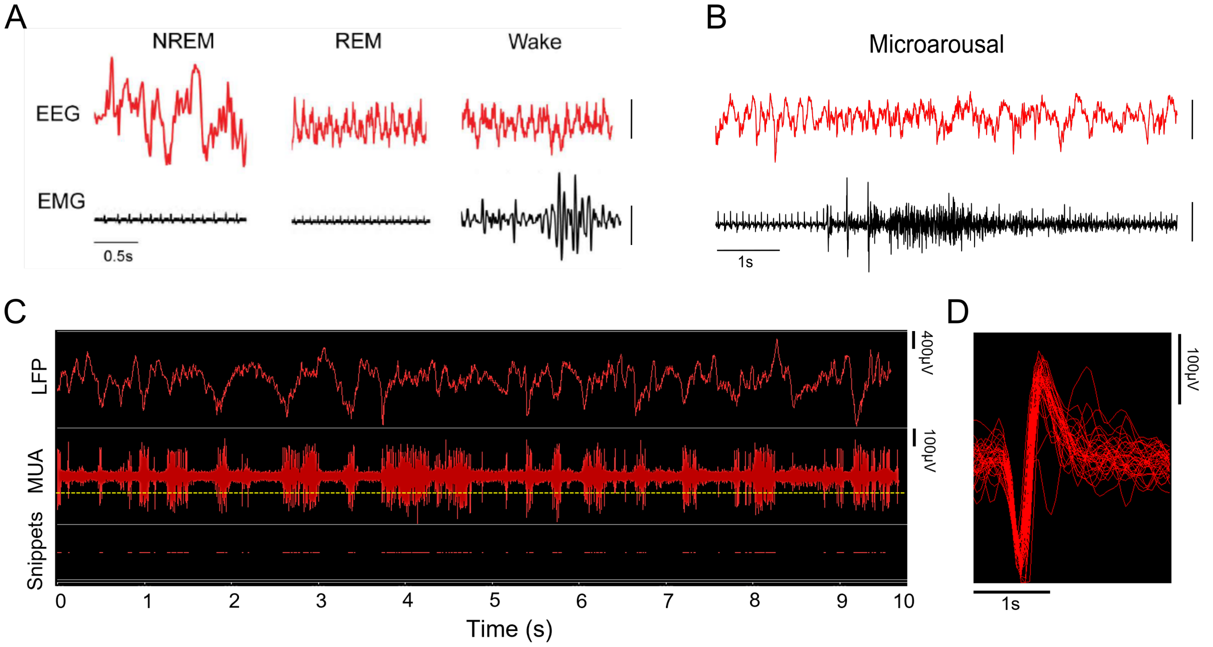

Supplementary Fig. S11 | Sleep stages, multi-unit activity and spike waveform. (A) Representative EEG signal segments from the frontal cortex (top) and the EMG (bottom) recorded in one mouse during different vigilance states (scale bar=250µV). (**B)** Representative EEG (frontal) and EMG traces recorded from one mouse during a microarousal. Microarousals were defined as transient periods of low voltage, high frequency oscillations in the EEG signals accompanied by elevated EMG tone, lasting ≥4s and ≤16s. **(C)** Representative LFP (top), MUA (middle) and snippets (timestamped spike waveforms) recorded simultaneously from S1 in one mouse during NREM sleep. The yellow dotted line represents the amplitude threshold used for spike acquisition. When the recorded voltage of the MUA crossed this threshold, 46 samples around the event (0.48 ms before, 1.36 ms after the threshold crossing) were extracted. **(D)** Corresponding waveforms of the action potentials recorded extracellularly. (*Note:* EEG: electroencephalography. EMG: electromyography. LFP: local field potential. MUA: multi-unit activity).

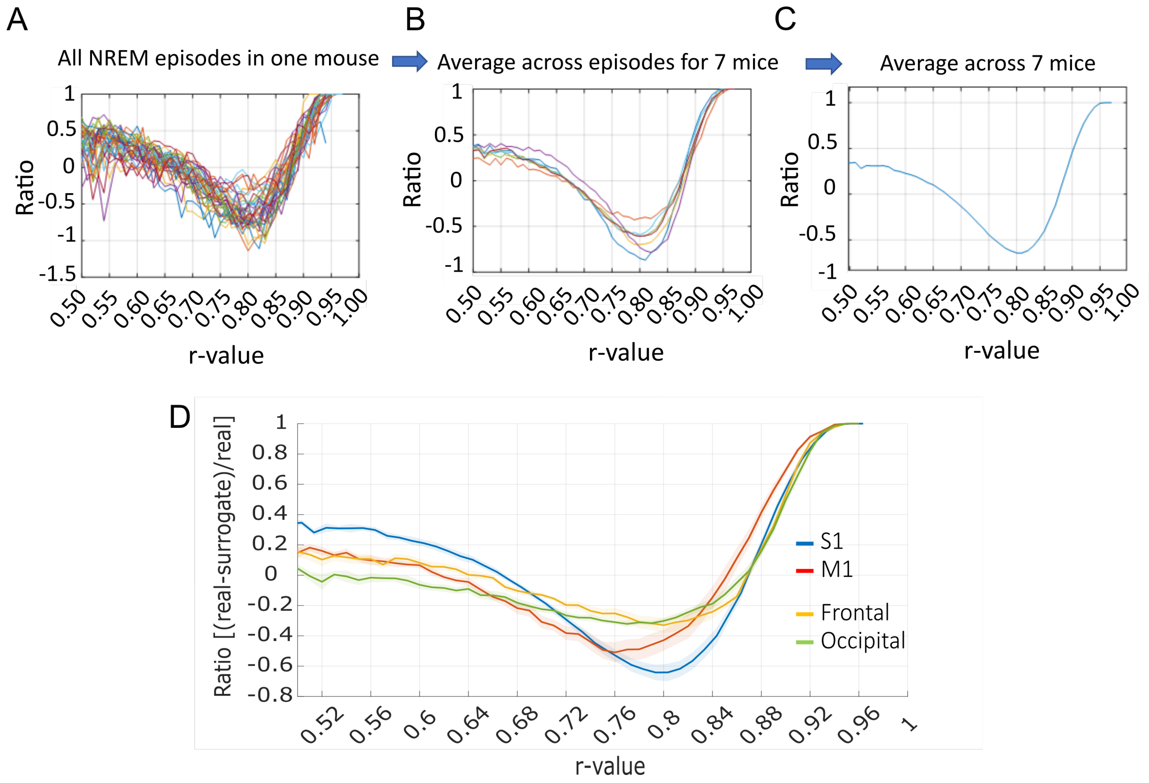

Supplementary Fig. S12 | Validation of the upper-threshold (rb). (A) The continuous distribution of r-values (negative logarithm proportional to damping constant) for a pole with frequency of 10-15Hz was calculated for every NREM episode and its corresponding surrogate signal. Here we show the rate (i.e. real signal – surrogate signal /real signal) between the r-value distributions in the real signals and their corresponding surrogates for all NREM episodes detected in one example mouse in the S1 LFP signal. Each colour represents an individual NREM episode. **(B)** Same as *A* but averaged across NREM episodes for n=7 mice. Each colour represents an individual mouse. **(C)** Same as *B* but averaged across mice. **(D)** Ratio between the r-value distributions in NREM episodes of real signals and respective surrogates recorded from intracortical channels (S1 and M1) and the EEG (frontal and occipital). Lines= average across NREM episodes and mice (n=7 per derivation), shaded area=SEM. (*Note:* S1: primary somatosensory cortex. LFP: local field potential. M1: primary motor cortex. EEG: electroencephalography).

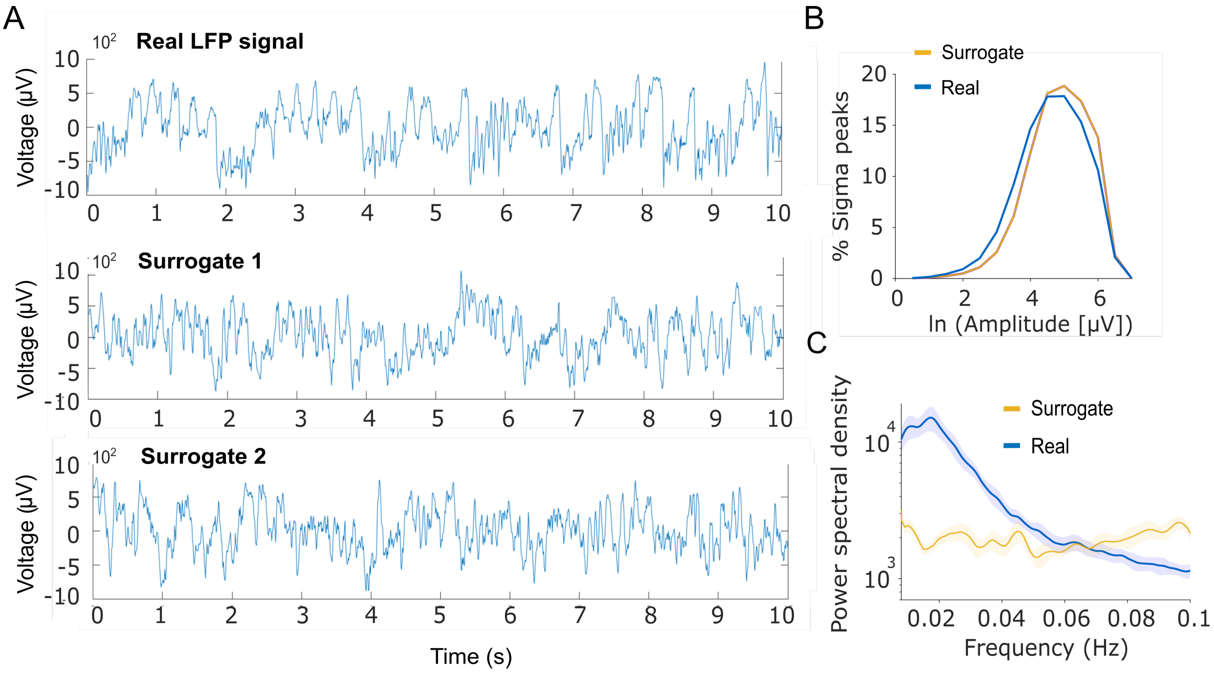

Supplementary Fig. S13 | Examples and properties of surrogate signals. (A) Example timeseries of a real LFP signal recorded from S1 in one mouse and two surrogate signals created based on an improved version of the IAAFT (Iterative Amplitude Adjusted Fourier Transform) algorithm. **(B)** Peak sigma (10-15 Hz) amplitude distribution calculated from a 10-minute segment of LFP real signal (in one mouse) and 19 respective surrogate signals. Shaded area= SEM. **(C)** Power spectral density ($\mu V^2/0.25Hz$) of the envelope (Hilbert transform) of filtered LFP signals (10-15 Hz) recorded from S1 and respective filtered (10-15 Hz) surrogates (n=19 per derivation per mouse). Figure shows mean and SEM (shaded area) across mice (n=7). (*Note:* LFP: local field potential. S1: primary somatosensory cortex. SEM: standard error of the mean).
